# Gain-of-Function Mutations in *ZIC1* Are Associated with Coronal Craniosynostosis and Learning Disability

**DOI:** 10.1016/j.ajhg.2015.07.007

**Published:** 2015-09-03

**Authors:** Stephen R.F. Twigg, Jennifer Forecki, Jacqueline A.C. Goos, Ivy C.A. Richardson, A. Jeannette M. Hoogeboom, Ans M.W. van den Ouweland, Sigrid M.A. Swagemakers, Maarten H. Lequin, Daniel Van Antwerp, Simon J. McGowan, Isabelle Westbury, Kerry A. Miller, Steven A. Wall, WGS500 Consortium, Peter J. van der Spek, Irene M.J. Mathijssen, Erwin Pauws, Christa S. Merzdorf, Andrew O.M. Wilkie

**Affiliations:** 1 Clinical Genetics Group, Weatherall Institute of Molecular Medicine, University of Oxford, John Radcliffe Hospital, Headington, Oxford OX3 9DS, UK; 2 Department of Cell Biology and Neuroscience, 513 Leon Johnson Hall, Montana State University, Bozeman, MT 59717, USA; 3 Department of Plastic Surgery, Erasmus MC, University Medical Center Rotterdam, PO Box 2040, 3000 CA Rotterdam, the Netherlands; 4 Developmental Biology and Cancer Programme, UCL Institute of Child Health, 30 Guilford Street, London WC1N 1EH, UK; 5 Department of Clinical Genetics, Erasmus MC, University Medical Center Rotterdam, PO Box 2040, 3000 CA Rotterdam, the Netherlands; 6 Department of Bioinformatics, Erasmus MC, University Medical Center Rotterdam, PO Box 2040, 3000 CA Rotterdam, the Netherlands; 7 Department of Pediatric Radiology, Erasmus MC, University Medical Center Rotterdam, PO Box 2040, 3000 CA Rotterdam, the Netherlands; 8 Computational Biology Research Group, Weatherall Institute of Molecular Medicine, University of Oxford, John Radcliffe Hospital, Headington, Oxford OX3 9DS, UK; 9 Craniofacial Unit, Department of Plastic and Reconstructive Surgery, Oxford University Hospitals NHS Trust, John Radcliffe Hospital, Oxford OX3 9DU, UK

## Abstract

Human *ZIC1* (zinc finger protein of cerebellum 1), one of five homologs of the *Drosophila* pair-rule gene *odd-paired*, encodes a transcription factor previously implicated in vertebrate brain development. Heterozygous deletions of *ZIC1* and its nearby paralog *ZIC4* on chromosome 3q25.1 are associated with Dandy-Walker malformation of the cerebellum, and loss of the orthologous *Zic1* gene in the mouse causes cerebellar hypoplasia and vertebral defects. We describe individuals from five families with heterozygous mutations located in the final (third) exon of *ZIC1* (encoding four nonsense and one missense change) who have a distinct phenotype in which severe craniosynostosis, specifically involving the coronal sutures, and variable learning disability are the most characteristic features. The location of the nonsense mutations predicts escape of mutant *ZIC1* transcripts from nonsense-mediated decay, which was confirmed in a cell line from an affected individual. Both nonsense and missense mutations are associated with altered and/or enhanced expression of a target gene, *engrailed-2*, in a *Xenopus* embryo assay. Analysis of mouse embryos revealed a localized domain of *Zic1* expression at embryonic days 11.5–12.5 in a region overlapping the supraorbital regulatory center, which patterns the coronal suture. We conclude that the human mutations uncover a previously unsuspected role for Zic1 in early cranial suture development, potentially by regulating *engrailed 1*, which was previously shown to be critical for positioning of the murine coronal suture. The diagnosis of a *ZIC1* mutation has significant implications for prognosis and we recommend genetic testing when common causes of coronal synostosis have been excluded.

## Introduction

Among the varied causes of craniosynostosis (premature fusion of one or more sutures of the skull vault), a monogenic etiology is most commonly identified in individuals with fusion of the coronal sutures, the major pair of transverse sutures crossing the vertex of the skull.^1^ Coronal synostosis, which can be present bilaterally (bicoronal) or unilaterally (unicoronal), affects approximately 1 in 10,000 children^2^ and is the type most commonly associated with an identifiable syndrome. Common monogenic disorders that characteristically present with coronal synostosis are Muenke (MIM: 602849) and Apert (MIM: 101200) syndromes, caused by localized gain-of-function mutations encoded by *FGFR3* (MIM: 134934) and *FGFR2* (MIM: 176943), respectively; Saethre-Chotzen syndrome (MIM: 101400) (*TWIST1* [MIM: 601622] haploinsufficiency); *TCF12*-related craniosynostosis (MIM: 600480 and 615314) (also a haploinsufficiency); and craniofrontonasal syndrome (MIM: 304110) (cellular interference involving variants in the X-linked *EFNB1* gene [MIM: 300035]).^3,4^

Even in the absence of an obvious syndromic diagnosis, a specific mutation can be identified in about 60% of individuals with bicoronal and 30% with unicoronal synostosis.^1,4^ The high monogenic load in coronal synostosis can be accounted for by the specific developmental origin of the coronal suture, which lies at an embryonic tissue boundary between neural-crest-derived frontal bone and mesoderm-derived parietal bone.^5,6^ Based on analysis of mouse models,^7^ coronal synostosis is frequently caused by disruption in the maintenance of the population of stem cells within the suture during early development (typically, embryonic days [E]12.5–14.5), caused, for example, by abnormalities in migration of neural crest cells^8^ or abnormal paracrine signaling through fibroblast growth factor receptors.^9,10^

An alternative possibility is that coronal synostosis could be caused by a primary failure of the suture to develop. Lineage tracing demonstrates that the cells of the future coronal suture originate from paraxial cephalic mesoderm at E7.5 and migrate laterally to locate above the developing eye.^11^ This region constitutes the supraorbital regulatory center and during E11.5–E13.5, cells from this zone migrate apically to form and populate the coronal suture.^6,11,12^ One of the genes characteristically expressed by these cells is *engrailed 1* (*En1*), a homolog of the *Drosophila engrailed* segment polarity gene. Mice with homozygous loss of *En1* function have generalized calvarial bone hypoplasia and persistent widening of the sutural gaps, which is associated with a posterior shift in the boundary between cells of neural crest and mesodermal origin.^11,13^ An orthologous mutation has not yet been described in humans.

Here, we report an additional genetic etiology for coronal synostosis, caused by heterozygous variants in the final exon of *ZIC1* (zinc finger protein of cerebellum 1 [MIM: 600470]), identified in four simplex case subjects and a three-generation pedigree. *ZIC1*, located on chromosome 3q25.1, belongs to a family of five genes encoding Zn-finger transcription factors, which are arranged as one unpaired and two paired paralogs in the human and mouse genomes;^14^*ZIC* genes are homologous to the *Drosophila* pair-rule gene *odd-paired*, which is required for activation of embryonic *engrailed* expression.^15^ Vertebrate ZICs have important roles in multiple developmental processes, including neurogenesis, left-right axis formation, myogenesis, and skeletal patterning.^16,17^ Heterozygous complete deletions of *ZIC1* were previously associated with Dandy-Walker malformation (DWM; hypoplasia and upward rotation of the cerebellar vermis and cystic dilatation of the fourth ventricle [MIM: 220200]);^18^ we now show that mutations affecting the highly conserved C terminus of the protein, which are likely to be associated with a gain of function, lead to a distinct phenotype of coronal suture fusion and learning disability. In addition to its previously established importance for neurogenesis,^19,20^ this work shows that *ZIC1* is required for normal coronal suture development. We find that murine *Zic1* is expressed in the supraorbital regulatory center, suggesting that this gene acts at a very early stage of coronal suture development,^7^ potentially (reflecting a similar epistatic relationship to that in *Drosophila*) by regulating *En1*.

## Subjects and Methods

### Subjects

The clinical studies were approved by Oxfordshire Research Ethics Committee B (reference C02.143), London Riverside Research Ethics Committee (reference 09/H0706/20), and the Medical Ethical Committee of the Erasmus University Medical Center Rotterdam (MEC-2012-140 and MEC-2013-547). Written informed consent to obtain samples for genetics research was obtained from each child’s parent or guardian. Venous blood was used for DNA extraction and fibroblast cultures were established from skin biopsies taken from scalp incisions during surgical intervention. Intracranial pressures in subject 1 were documented by 24–48 hr direct recording with an intraparenchymal Codman Microsensor.^21^ The screening panel comprised samples from 307 individuals with syndromic or non-syndromic craniosynostosis. All DNA samples were previously tested for mutation hotspots in *FGFR2*, *FGFR3*, *TWIST1*, and *TCF12*.^3,4^ Significant chromosome aneuploidy in individuals with *ZIC1* mutations was excluded by karyotyping and/or array comparative genomic hybridization. Where necessary, correct biological relationships were confirmed by segregation analysis of a panel of 13 microsatellites (*D1S2868*, *D3S1311*, *D4S403*, *D5S2027*, *D6S1610*, *D7S519*, *D9S158*, *D10S548*, *D11S898*, *D13S1265*, *D14S280*, *D16S415*, and *D18S474*).

### Whole Genome/Exome Sequencing and Mutation Screening of *ZIC1*

Whole genome sequencing (WGS) of the male proband subject 1 and his parents was performed as part of the WGS500 clinical genome sequencing initiative.^22^ In brief, 3–5 μg DNA was used to prepare libraries for 100 bp paired-end sequencing to generate a mean coverage of 30× using the Illumina HiSeq2000 platform. Sequence reads were mapped to the human reference GRCh37d5 using Stampy (v1.0.12–1.0.22) and variants called with Platypus (v.0.2.4).^23,24^ To identify de novo mutations, we prioritized variants within coding regions that were called as absent in both parents and in dbSNP135, generating a list of 203 variants in 177 genes, of which 39 were classified as protein altering. Visualization of the trio read alignments revealed a single bona fide change in *ZIC1* (12 of 27 reads), which was absent in both the paternal (19 reads) and maternal (31 reads) samples; the other 38 variants were either in fact present in one of the parents or were artifactual (Table S1). Possible recessive inheritance was analyzed with an in-house perl script to list homozygous, compound heterozygous, and hemizygous X chromosomal variants in subject 1, with a frequency cut-off of 0.003 in either 1000 Genomes or Exome Variant Server; variants in two genes fitted the criteria (Table S1). Whole genome sequencing of genomic DNA from four subjects in family 5 (affected: 5:II.2, 5:III.3, 5:III.6; unaffected: 5:II.3) was performed by BGI Complete Genomics.^25,26^ Filtering based on a list of genes mutated in craniosynostosis identified a predicted missense substitution encoded by *ZIC1*, present only in the three affected individuals. Exome sequencing of subject 3 was performed on genomic DNA (extracted from whole blood) using an Agilent SureSelect Human All Exon Kit (v.5; 50 Mb) on the Illumina HiSeq2000 platform. Reads were mapped to hg19 with Novoalign (Novocraft Technologies) and variants called with SAMtools and annotated by ANNOVAR.

To investigate further the significance of the *ZIC1* mutations, primers were designed for amplification of genomic DNA (GenBank: NT_005612.17) and cDNA (GenBank: NM_003412.3), for multiplex ligation-dependent probe amplification (MLPA) analysis, and for deep sequencing (Table S2, which provides details of experimental conditions). Variant screening of all three exons of *ZIC1* was performed by dideoxy sequencing on PCR amplification products from genomic DNA by BigDye Terminator v3.1 (Applied Biosystems). Copy-number variation was analyzed by MLPA using probes to each exon, according to the manufacturer’s instructions (MRC Holland). RNA was extracted from fibroblasts (Trizol, Invitrogen), cDNA synthesized with RevertAid first strand cDNA kit (Thermo Scientific), and the samples analyzed by agarose gel electrophoresis after digestion with BfaI. To quantify the proportions of wild-type to mutant allele in cDNA, an amplification product spanning exons 2–3 was used as a template for PCR to add Ion Torrent P1 and A adapters, and the resulting product was purified with AMPure beads (Beckman Coulter). Emulsion PCR and enrichment were performed with the Ion PGM Template OT2 200 Kit (Life Technologies) according to the manufacturer’s instructions and sequencing of enriched templates performed on the Ion Torrent PGM (Life Technologies) for 125 cycles with the Ion PGM Sequencing 200 kit v2. Data were processed with Ion Torrent platform-specific pipeline software v.4.2.1.

### *Xenopus* Assays

All experiments using *Xenopus* were approved by the Institutional Animal Care and Use Committee of Montana State University. *Xenopus* full-length *zic1*^27^ and *zic1ΔC*^28^ cDNA constructs were described previously (*zic1ΔC* was originally termed *oplΔC*). The *zic1ΔC2* construct was made by PCR amplification of the portion of *zic1* cDNA encoding the N terminus and zinc finger domains, including four amino acids of the C-terminal region, followed by cloning into the EcoR1 and Xba1 sites of pCS2+ATG.^27^ The human *ZIC1* cDNA pCR4-Topo-ZIC1 (ThermoFisher) was subcloned into pcDNA3 and six different nucleotide substitutions—c.895G>T (p.Glu299^∗^), c.1163C>A (p.Ser388^∗^), c.1198G>C (p.Gly400Arg), c.1204G>T (p.Glu402^∗^), c.1240A>G (p.Thr414Ala), and c.1309_1310GC>TA (p.Ala437^∗^)—were introduced by PCR mutagenesis using the primer sequences and experimental conditions provided in Table S2. The human *ZIC1* constructs were subsequently digested with EcoR1 and Xba1 and ligated into the pCS2+ plasmid. Capped sense RNAs for microinjection were synthesized from the *Xenopus* and human pCS2+ constructs by SP6 transcription of NarI linearized plasmids. *Xenopus laevis* eggs were collected and fertilized as previously described^28^ and embryos were staged according to Nieuwkoop and Faber.^29^ Embryos at the two-cell stage were injected into a single cell with 200 pg sense RNA synthesized from cDNA constructs, together with 25 pg *lacZ* RNA as tracer. After β-galactosidase staining,^30^ wild-type embryos were bleached by exposing the embryos to fluorescent light in hybridization buffer containing 1% H_2_O_2_. Expression of *en-2* was determined in neurula stage 15–17 albino and wild-type embryos by in situ hybridization^31^ with digoxygenin-labeled antisense RNA *en-2* probe as described.^32^ An anti-digoxygenin alkaline phosphatase-conjugated antibody (Roche) and the alkaline phosphatase substrate NBT/BCIP (Fisher Scientific) were used for color detection. Embryos were scored double-blind to determine changes in *en-2* expression in comparison to the uninjected side. Results using wild-type and mutant constructs were compared by Fisher’s exact tests with Bonferroni correction for multiple comparisons (n = 9).

### RNA In Situ Hybridization of Mouse Embryos

Experimental procedures were performed in accordance with UK Animals (Scientific Procedures) Act, 1986 (PPL 70/7194). For whole-mount embryo in situ hybridization, embryos were dissected, fixed overnight in 4% paraformaldehyde in phosphate-buffered saline, and dehydrated through graded methanol solutions. Non-radioactive RNA in situ hybridization was performed as described^33^ before vibratome sectioning. RNA probes for *Zic1*^34^ and *En1*^35^ were digoxygenin labeled with the In Vitro Transcription kit (Roche Applied Science) followed by anti-digoxygenin-AP antibody (1:1,000) (Roche Applied Science) and NBT/BCIP (Sigma) staining to detect the hybridization signals.

## Results

### Identification of *ZIC1* Mutations

**Figure 1:**
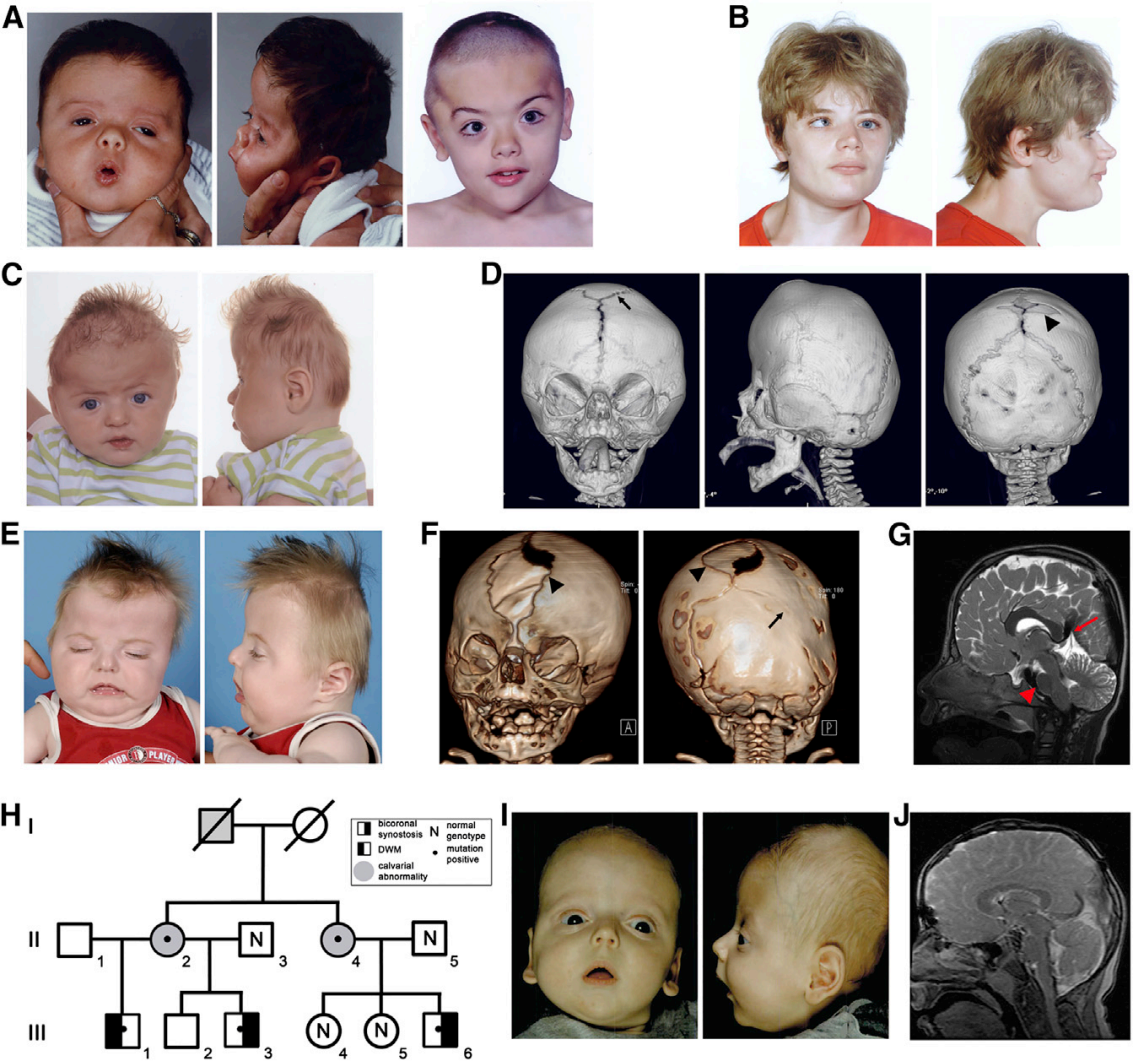
Clinical and Radiological Phenotype of Individuals with *ZIC1* Mutations (A) Subject 1, age 7 weeks (left) and 8 years (right). (B) Subject 2, age 23 years. (C) Subject 3, age 5 months. (D) CT head scan of subject 3 (age 5 months) showing bicoronal synostosis, a large wormian bone (arrow) in the position of the anterior fontanelle, and an ossification defect in the sagittal suture (arrowhead). (E) Subject 4, age 7 months. (F) CT head scan of subject 4 (age 2.5 months). Note asymmetric skull shape associated with bilateral coronal and right lambdoid suture fusion (arrow). Sections of the metopic and sagittal sutures remain widely patent (arrowheads). (G) MRI brain scan (T2 image) of subject 4, age 16 months. Note short, broad corpus callosum, peaked tentorium cerebelli (arrow), and hypoplasia of the pons (arrowhead), cerebellar vermis, and cerebellar hemispheres. (H) Pedigree of family 5. (I) Subject 5:III.6, age 2 months. (J) MRI brain scan (T2 image) of subject 5:III.6, age 13 years. Abnormal features show a similar pattern to those present in subject 4.

The proband (subject 1) presented at birth with severe brachycephaly (Figure 1A), which was shown by three-dimensional computed tomographic reconstruction (3D-CT) to be caused by bicoronal synostosis. He required three major craniofacial surgical procedures (at the ages of 7 months, 2.4 years, and 4.8 years), the latter two because of raised intracranial pressure. In addition he had autistic traits and moderate-severe learning disability, features that are rarely associated with coronal synostosis. Genetic testing for mutations known to be associated with coronal synostosis was negative; his phenotype is summarized in Table 1 and more detailed descriptions of all subjects are provided in the Supplemental Data (Case Reports).

**Figure 2:**
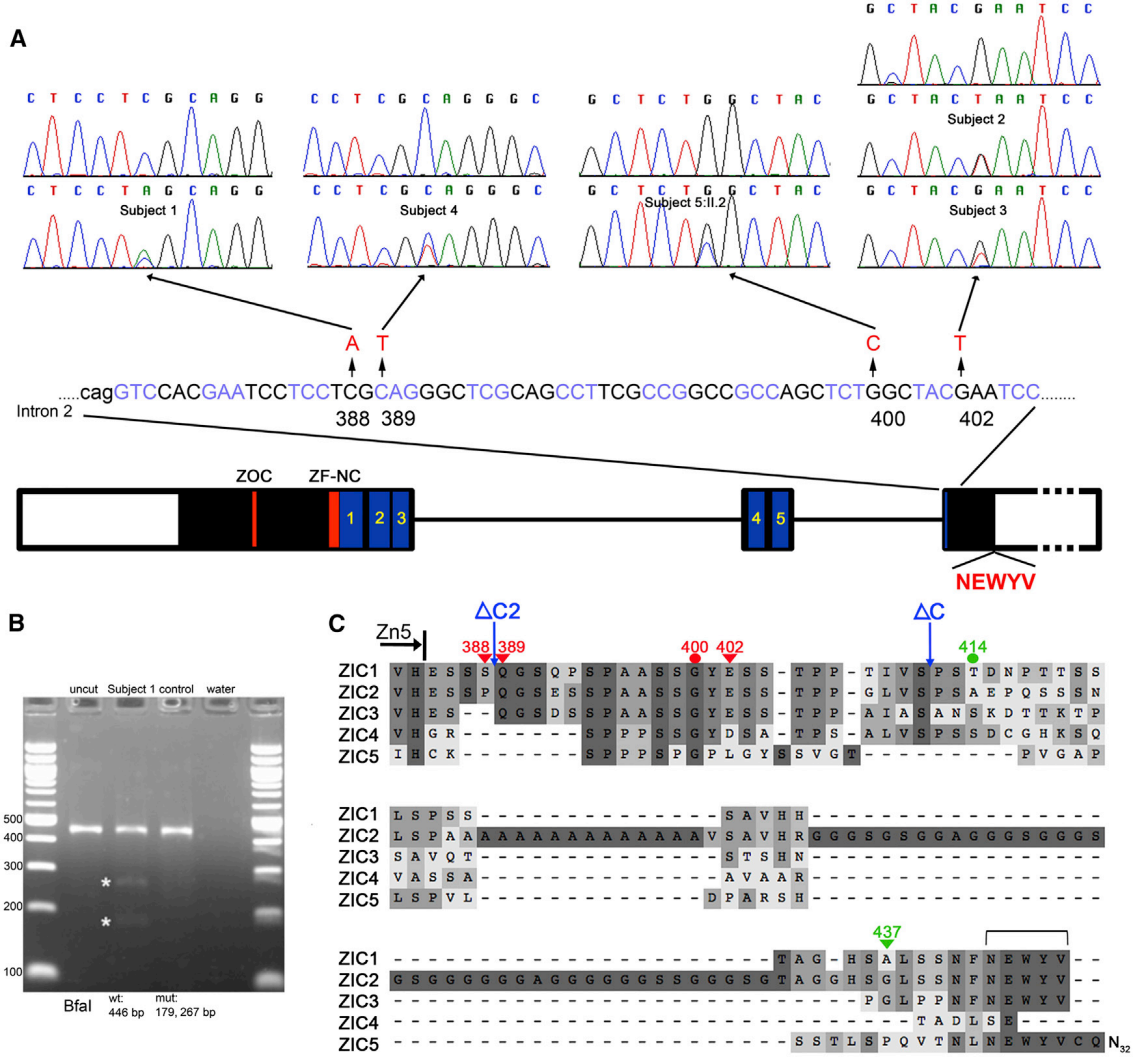
Molecular Genetic Analysis of Individuals with *ZIC1* Mutations (A) Cartoon showing exon organization (white boxes denote non-coding regions) and previously identified conserved domains (Zic opa conserved motif [ZOC], zinc finger N-flanking conserved region [ZF-NC], and five zinc fingers [1–5, blue boxes]) of human *ZIC1*.^16^ Also indicated is the C-terminal NEWYV motif conserved in all family members except *ZIC4*. Above the cartoon are the positions of the five independent *ZIC1* mutations described in this report, and dideoxy-sequence traces showing comparison of normal sequence (above) and mutant sequence (below). Note, in the case of subject 3, the mutation was not evident in the DNA sample (sourced from scalp fibroblasts; not shown) originally analyzed; however, in the exome sequence of DNA sourced from blood of the same individual, 63 of 183 (34%) reads showed the c.1204G>T mutation, which is also readily apparent on the dideoxy sequence. The relative heights of mutant and wild-type peaks differ between samples from subject 3 and subject 2, who is constitutionally heterozygous for the identical mutation, corroborating that in subject 3 the mutation is present in high-level mosaic state. (B) Agarose gel analysis of *ZIC1* cDNA obtained from RNA extracted from scalp fibroblasts of subject 1 and digested with BfaI. The fragments yielded by digestion of the mutant allele are indicated with asterisks. (C) Amino acid sequence encoded by 3′-terminal exon of *ZIC1* and comparison with the paralogous human proteins ZIC2-ZIC5, showing conservation including the NEWYV motif (bracket). The end of the fifth zinc finger (Zn5) is shown above the sequence, as are the positions of the four different pathogenic variants (red symbols) (triangle, nonsense; circle, missense) identified in this study. The positions at which the *Xenopus* constructs *zic1ΔC2* and *zic1ΔC* are truncated, relative to the human sequence, are indicated by blue arrows (note *zic1ΔC2* is equivalent to p.Gln389^∗^). Additional human constructs tested in the *Xenopus* assay are indicated by green symbols.

We undertook WGS of the parent-child trio and analyzed the data for variants consistent with either (1) autosomal or X-linked recessive inheritance or (2) a new dominant mutation (Table S1). After filtering, variants in two genes were consistent with the recessive disease model, but these genes, *CNGA3* (MIM: 600053) and *NEB* (MIM: 161650), are associated with achromatopsia 2 (MIM: 216900) and nemaline myopathy 2 (MIM: 256030), respectively, disorders without a craniofacial phenotype, and thus were not considered further. The single dominant candidate was a heterozygous c.1163C>A mutation in *ZIC1* (GenBank: NM_003412.3), predicting the nonsense change p.Ser388^∗^. This variant was absent in the parental samples and was confirmed by dideoxy sequencing (Figure 2A).

The significance of this de novo *ZIC1* mutation was initially uncertain. Contiguous heterozygous deletions of *ZIC1* and its adjacent paralog *ZIC4* were previously described in DWM,^18^ although a few deletion cases have lacked this characteristic phenotype.^36^ Review of the CT scan in subject 1 showed no brain malformation, and on a later magnetic resonance imaging (MRI) scan, only minor abnormalities of configuration of the ventricles and corpus callosum were evident (not shown).

Nonsense mutations of *ZIC1* have not previously been reported, but the location of the nucleotide substitution in the terminal (third) exon (Figure 2A) predicted that it would escape nonsense-mediated decay.^37^ We digested *ZIC1* cDNA generated from scalp fibroblasts with the restriction enzyme BfaI, which cuts the mutant allele, and found that the expected mutant fragments were readily visualized (Figure 2B); we then used deep sequencing for accurate quantification and found that 7,175 of 13,088 reads (55%) represented mutant alleles, confirming escape from nonsense-mediated decay (not shown). A prematurely truncated translation product could be associated with dominant-negative or gain-of-function mechanism, distinct from the previously described deletions.^37^ Support for a gain-of-function mechanism was provided by previously published work on the *Xenopus zic1* ortholog, formerly known as *opl* (*odd-paired*-like), in which it had been shown that a cDNA construct, *zic1ΔC*, containing a shorter truncation missing the C-terminal 36 amino acids (see Figure 2C), had enhanced activity compared to full-length cDNA, in transactivation, and *Xenopus* animal cap, and other in vivo assays.^27,38,39^

To search for further evidence that *ZIC1* mutations cause craniosynostosis, we screened a panel comprising 307 unrelated subjects with synostosis affecting any combination of sutures (including 45 and 112 with exclusively bilateral and unilateral coronal synostosis, respectively) and for whom no genetic diagnosis had been made. Initially we identified a single heterozygous nonsense mutation in this panel (subject 2: c.1204G>T encoding p.Glu402^∗^); neither parent had the mutation, indicating that it had arisen de novo (sample relationships were confirmed by microsatellite analysis). We did not identify any *ZIC1* copy-number changes in this craniosynostosis panel by MLPA (data not shown). Later, we discovered by exome sequencing that a second individual included on the panel (subject 3) had the identical mutation, but present in mosaic state (see legend to Figure 2A for details). Strikingly, review of the phenotypes of subjects 2 and 3 revealed that both had bicoronal synostosis with severe brachycephaly (Figures 1B–1D); in addition, both had learning disability, which was milder in subject 3 who had the mosaic mutation. Review of CT brain scans showed that subject 2 had agenesis of corpus callosum and dilated lateral ventricles, but neither subject 2 or 3 had DWM (not shown).

In an attempt to further replicate these findings, we examined DNA samples collected at a second craniofacial unit (Rotterdam), specifically where the combination of both coronal synostosis and significant learning disability was present. Only three samples were available for analysis, which reflects the rarity of this combination of phenotypes; remarkably, however, dideoxy sequencing showed that one of these samples harbored a heterozygous nonsense mutation in *ZIC1* (c.1165C>T encoding p.Gln389^∗^) at the codon adjacent to that affected in subject 1 (Figure 2A). This child had presented with bicoronal and unilateral lambdoid synostosis (Figures 1E and 1F) and had significant learning problems (Table 1). An MRI scan identified several cerebral anomalies including a short corpus callosum, mildly enlarged lateral ventricles, peaked tentorium, hypoplastic pons, and cerebellum with prominent cerebellar folia and enlarged foramen magnum with signal void near the cervical cord (Figure 1G).

The final family (family 5) consisted of six affected individuals in three generations (Figure 1H). Two cousins (subjects 5:III.3 and 5:III.6) had bicoronal synostosis (Figures 1I and 1J) and a further individual (subject 5:III.1, a half-brother of 5:III.3) had a DWM but no craniosynostosis (not shown). All three, and their respective mothers (5:II.2 and 5:II.4), had mild learning disability. Whole genome sequencing of four individuals (subjects 5:II.2, 5:II.3, 5:III.3, 5:III.6) identified a heterozygous variant in *ZIC1*, c.1198G>C encoding p.Gly400Arg, present in the three affected individuals (5:II.2, 5:III.3, 5:III.6) but not in the unaffected spouse (5:II.3). These results were confirmed by dideoxy sequencing, which showed that the variant was also present in 5:III.1 (the individual with DWM) and in 5:II.4 (the obligate transmitting mother) but not in her two unaffected children (5:III.4 and 5:III.5). This variant is absent from more than 120,000 alleles in the Exome Aggregation Consortium (ExAC). The combination of phenotypic features, the segregation of the variant, its location in the region where the previously identified nonsense mutations all clustered (Figure 2A), and absence in large databases of variation suggest that this variant is causative of the phenotype.

### Functional Consequence of *ZIC1* Mutations in *Xenopus* Embryo Assay

The function of the *Xenopus zic1* ortholog has been studied in detail, where it was shown to act together with Pax3 as a key transcription factor required for the initiation of neural crest formation.^40^ In *Xenopus* embryos, a signaling cascade has been proposed in which inhibition of bone morphogenetic proteins (Bmps) activates *zic1*, which in turn activates members of the Wnt family including *wnt1*, which in turn activates the transcription factor *engrailed-2* (*en-2*).^27,38^ A previously designed assay had shown that injection of *zic1ΔC* RNA encoding a truncated ZIC1 into a single cell of 2-cell stage embryos led to increased *en-2* expression in stage 15–17 *Xenopus* embryos, whereas injection of wild-type *zic1* construct had no effect.^27^ We next examined the activity of human *ZIC1* and various mutant constructs with this assay.

**Figure 3:**
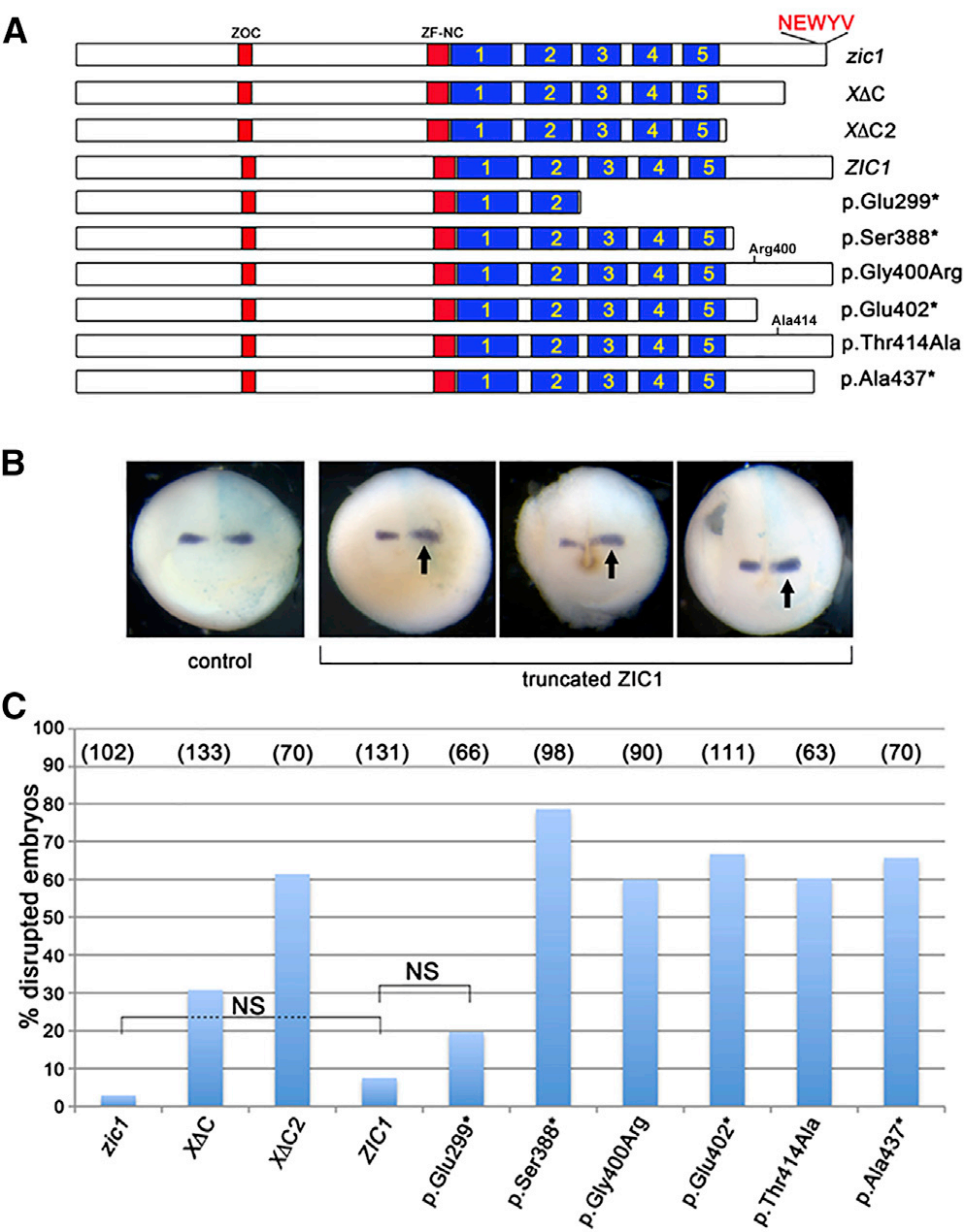
Analysis of Consequences of *ZIC1* Mutations in *Xenopus* Embryos (A) Cartoon showing the structure and nomenclature of the cDNA constructs used in the experiment. The five zinc finger domains are highlighted in blue. (B) *Xenopus en-2* expression after microinjection of *ZIC1* construct RNA (right side of each embryo). Arrows indicate widened, increased, or shifted *en-2* expression in three representative embryos. (C) Quantification of effects of *ZIC1* mutations on *en-2* expression. The numbers of embryos used in each experiment are indicated in parentheses. Statistical comparisons between full-length *Xenopus zic1* and human *ZIC1* and between *ZIC1* and *ZIC1*-p.Glu299^∗^ were not significant (NS), whereas comparisons between all other mutants and corresponding full-length constructs were highly significant (p < 10^−8^).

In initial experiments, we replicated the previously reported results of the *Xenopus* assay. Although very few (3%) embryos were disrupted by injection of the wild-type *zic1* construct, this increased to 31% using the previously published *zic1ΔC*. Moreover, a construct deleting almost the entire region C-terminal to the zinc fingers (*zic1ΔC2*; Figure 2C) showed an even higher proportion (61%) of disrupted embryos (Figure 3). Using constructs encoding human ZIC1, injection of full-length *ZIC1* RNA yielded only 8% disrupted embryos, but this was increased to 79% and 68% with constructs corresponding to two of the observed truncations, p.Ser388^∗^ (subject 1) and p.Glu402^∗^ (subjects 2 and 3), respectively. Strikingly, a similar magnitude of effect (66%) was found with the most C-terminal truncation construct (p.Ala437^∗^, which does not correspond to an observed mutation), highlighting the importance of the terminal 11 amino acids, which includes a 5-amino-acid motif (NEWYV) of unknown function that is conserved in human ZIC2, ZIC3 (isoform A), and ZIC5 (Figure 2C) but is not present in any other human protein. Two constructs encoding missense substitutions were studied with the same assay: p.Gly400Arg present in family 5 and p.Thr414Ala, corresponding to a rare SNP (dbSNP rs143292136), present in 104/121,380 alleles in ExAC, which we had identified in a child with bicoronal synostosis and her unaffected father (data not shown); 60% of embryos were disrupted in both cases. Although producing a slightly milder effect than the nonsense mutations, these results suggest that residues in the C-terminal domain in addition to the NEWYV motif contribute to function. Importantly, using a truncated control construct (p.Glu299^∗^) missing the last three of the five zinc fingers, a much lower proportion of embryos (20%) was disrupted, which did not differ significantly from the full-length *ZIC1* RNA (Figure 3C). This indicates that intact zinc fingers are required to induce consistently abnormal *en-2* expression in this assay.

### *Zic1* Expression in Mouse Embryos

**Figure 4:**
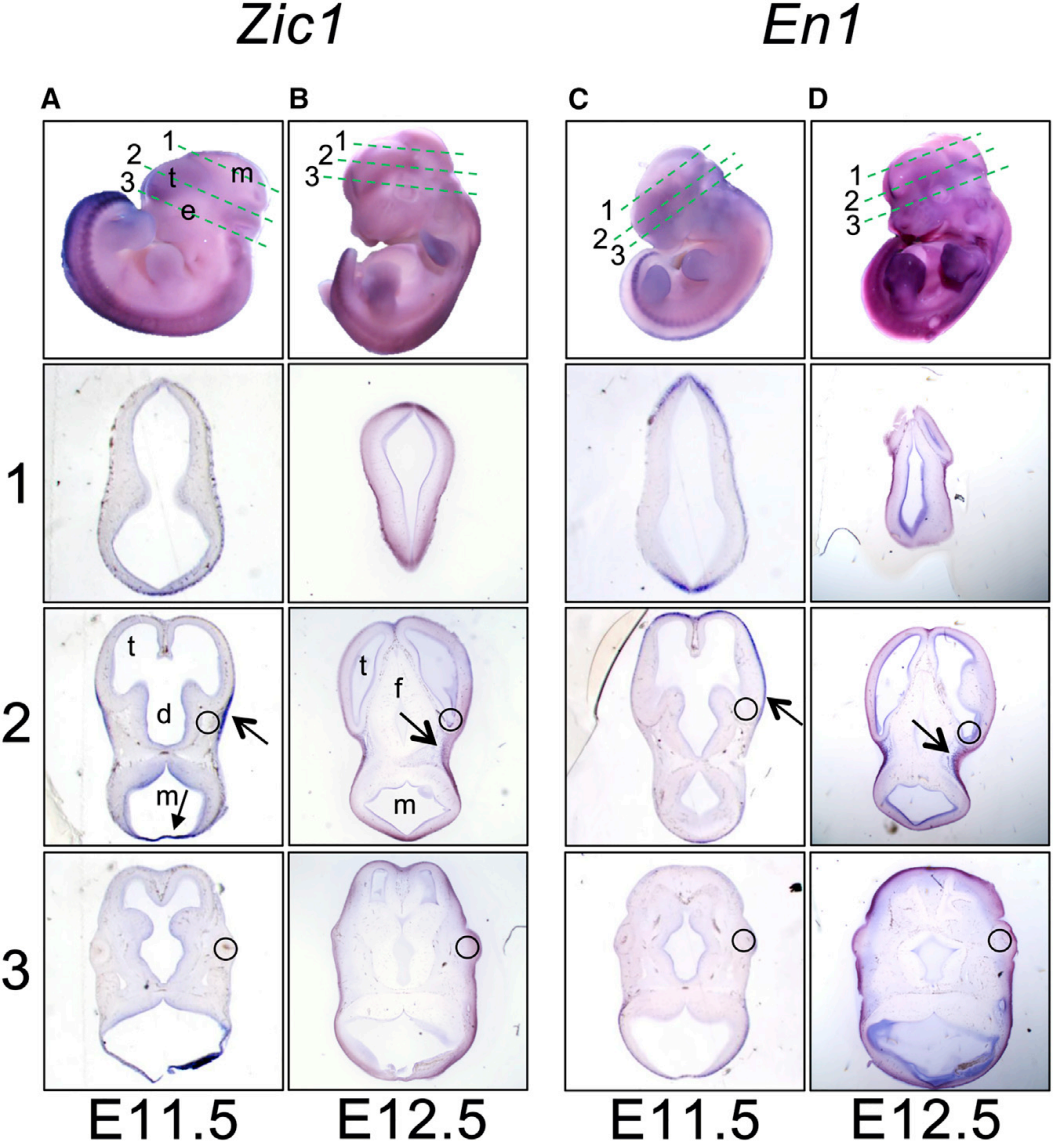
Expression of *Zic1* in E11.5–E12.5 Mouse Embryos Analyzed by RNA In Situ Hybridization and Comparison with *En1* Panels show comparison of *Zic1* (A, B) and *En1* (C, D) expression at E11.5 (A, C) and E12.5 (B, D). In each case the top panel shows the whole embryo and plane of sections 1, 2, and 3, which are illustrated in the three respective lower panels (e, eye; t, telencephalon; d, diencephalon; m, mesencephalon; f, forebrain; circles indicate relative position of the eye). Note strong expression of *Zic1* in supra-orbital region (open arrow) at E11.5; other areas of expression are midbrain-hindbrain boundary (closed arrow), neural tube, and limb mesenchyme (A). At E12.5 *Zic1* expression is seen mainly in the cephalic mesenchyme, just posterior to the eye (B). By comparison, at E11.5 the expression of *En1* expression is relatively weak in the supraorbital region (stronger expression is seen in the apical ectodermal ridge of the limb and in the somitic mesoderm). By E12.5 *En1* expression has increased in the cephalic mesoderm.

Of note in the above experiments, the effect of the ZIC1 C-terminal mutants was to increase the expression of the *Xenopus* target gene *en-2* (Figures 3B and 3C). Given previous evidence that the paralogous gene *En1* is critical for early biogenesis of the murine coronal suture,^11^ we asked whether *Zic1* might also be expressed in relevant cells. Although the neural pattern of *Zic1* expression^20,41^ and loss-of-function phenotypes^19,20^ were previously described in the mouse, no evidence has linked *Zic1* expression to coronal suture development. Therefore, we analyzed expression patterns of *Zic1* in embryonic mouse heads between E11.5 and E17.5. Between E11.5 and E12.5, a distinct domain of *Zic1* expression was observed in the supraorbital region and cephalic mesoderm, which appeared to precede and partly overlap *En1* expression (Figure 4). By contrast, no *Zic1* expression was observed in the calvaria at E14.5 and E17.5 (not shown).

## Discussion

Given the homology to one of the classical early patterning genes of the *Drosophila* embryo, the function of the vertebrate Zic family has been the focus of sustained interest. Highlighting their importance for development, mutations in the related genes *ZIC2* (MIM: 603073) and *ZIC3* (MIM: 300265) were previously described in holoprosencephaly (MIM: 609637)^42^ and X-linked visceral heterotaxy (MIM: 306955),^43^ respectively. In the case of *ZIC1*, the observation that heterozygous deletions in tandem with *ZIC4* cause DWM^18,36^ has supported work in *Xenopus*^27,38,40^ and mouse,^20,41^ indicating key roles for the Zic1 ortholog in neurogenesis (in the mouse, deficiency of *Zic1* and *Zic4* contribute additively to cerebellar hypoplasia).^20^ Our data now highlight a previously unsuspected subsidiary role for ZIC1 in early patterning events in the coronal suture.

The nonsense mutations of *ZIC1* present in subjects 1 to 4 all arose de novo (post-zygotically in the case of subject 3, who has an attenuated phenotype) and are associated with consistent features comprising bicoronal synostosis, moderate to severe learning disability, and subtle abnormalities in brain anatomy including variable deficiency of the corpus callosum and abnormal conformation of the ventricles and posterior fossa. The progressive scoliosis in two of these individuals is likely to be causally related to the mutation, because vertebral and thoracic defects were also observed in mouse mutants homozygous for the null mutation in *Zic1*.^44^ In family 5, a heterozygous missense variant p.Gly400Arg, present in the same C-terminal region of the ZIC1 protein, segregated through three generations in six affected individuals. The phenotype in this family was variable, with two individuals having documented bicoronal synostosis and another a DWM. Associated learning disabilities were less severe than in subjects with truncations. Interestingly, MRI scans showed that several individuals from this family not formally diagnosed with DWM nevertheless had more subtle abnormalities in the conformation of the posterior fossa (Figure 1J). Hence the cerebral features were reminiscent of, but milder than, the malformations described in the previous cases with contiguous *ZIC1*-*ZIC4* deletions. In contrast to many craniosynostosis syndromes, no diagnostic limb anomalies were apparent in any of the affected individuals.

In assessing the association of *ZIC1* mutations with craniosynostosis, two features are particularly striking: the highly localized distribution of the mutations and the severity of the phenotype. All five mutations predict protein alterations within a 15-residue stretch encoded by the final exon. The occurrence of distinct phenotypes and/or patterns of inheritance associated with truncations localized to the terminal exon is a well-recognized indication that escape from nonsense-mediated decay might be responsible.^37^ Indeed, we were able to demonstrate that the transcript carrying the nonsense mutation was stable in a fibroblast cell line from the individual (subject 1) with the most N-terminal truncation (Figure 2B). The qualitative difference in phenotype associated with heterozygous truncating mutations, compared to previously reported heterozygous deletions,^18,36^ supports a gain-of-function mechanism (see also below). In the four case subjects with truncating mutations, the coronal synostosis was always bilateral and associated with marked brachycephaly (Figures 1A–1G), consistent with a severe, early effect on cranial suture formation; localized sutural ossification defects also occurred frequently. Craniosynostosis accompanied by DWM is rare, although an association with sagittal synostosis was reported previously.^45^

To investigate the mechanism by which these mutations could disturb coronal suture biogenesis, we first explored their functional effect in a previously established *Xenopus* assay. Injection of several constructs truncated at different positions in the C-terminal region led to disrupted *en-2* expression in a majority of treated embryos, provided that the zinc finger domain remained intact (Figure 3). Mostly the disrupted expression pattern involved combinations of upregulation, shifting, and expansion of *en-2* expression (for examples see Figure 3B), although with the larger truncations retaining all Zn fingers, some embryos showed reduced *en-2* expression. The *zic1ΔC* construct has been used previously in *Xenopus* to obtain enhanced biological responses to *zic1* in several assays in which sensitization of the ectoderm to Bmp inhibition led to activation of genes expressed in the neural crest and neural tube; this was interpreted as showing that the C-terminal region has negative regulatory activity.^38^ The biological basis of this activity remains unknown; based on the enhanced *en-2* expression associated with both the very C-terminal truncation p.Ala437^∗^ and the two missense substitutions, this might involve protein interaction over several parts of the ZIC1 C-terminal region, which shows several patches that are conserved between multiple ZIC paralogs (Figure 2C). Although the *Xenopus* assay supports the genetic evidence that the c.1198G>C (p.Gly400Arg) substitution is causative of the phenotype in family 5, a cautionary note is provided by the finding that p.Thr414Ala also disrupted *en-2* expression, because this variant occurs too frequently (at 1 in 1,167 alleles) to be penetrant for craniosynostosis in more than a small proportion of individuals who carry this variant.

In a second approach to understand the pathogenic mechanisms, we asked whether the pattern of *Zic1* expression was consistent with a specific role in coronal suture biogenesis. We found a previously undescribed, transient zone of *Zic1* expression in the supraorbital region at E11.5 (Figure 4) that is spatially and temporally overlapping with that of *En1* (Figure 4) and is consistent with an early instructive role for *Zic1* in the supraorbital regulatory center; combining these observations with the finding of increased *en-2* expression driven by mutant ZIC1 constructs in the *Xenopus* experiments, we propose that the coronal synostosis phenotype associated with the human mutations might be attributable to alteration of *EN1* expression in the supraorbital regulatory center, thus disrupting the patterning of the coronal suture at a very early stage in its development. The induction of *engrailed* expression by *ZIC1* orthologs is well characterized in both *Drosophila*^15,46^ and *Xenopus*^38^ and is thought to act through the *Wnt* signaling pathway.^27^ A further component of this signaling network is likely to be *Lmx1b*, which encodes a LIM-homeodomain protein and is upregulated in early neural crest of *Xenopus*.^47^ In the mouse, *Lmx1b* is prominently expressed in the supraorbital region at E11.5, and homozygous mutants have severely abnormal cranial sutures.^48^ In humans, heterozygous mutations in *LMX1B* usually cause nail-patella syndrome (MIM: 161200),^49^ but a specific missense mutation in the N-terminal arm of the homeodomain has been associated with craniosynostosis.^1^

Putting the evidence together, we propose that the *ZIC1* mutations we have described serendipitously uncover an important role for this transcription factor in early lineage commitment at the supraorbital regulatory center^7^ and that *En1* is likely to represent a key target gene. This will stimulate further work to define more precisely the role of *Zic1* in the coronal suture and to delineate the function of the conserved C-terminal domain. In *ZIC2*, constructs with holoprosencephaly-associated mutations C-terminal to the zinc fingers showed variable loss of transactivation activity,^50^ whereas in *ZIC3*, the single missense mutation beyond the zinc fingers reported to date (in a simplex case subject with congenital heart disease) was uniquely associated with increased transactivation activity.^51^ Collectively these data highlight the distinct properties of the C-terminal domain of ZIC proteins. The sequence conservation identified in this region (Figure 2C) suggests that a shared but currently unexplored mechanism exists for their regulatory function. Finally, although *ZIC1* mutations are rare because the genetic target is localized, the complications for intellectual and skeletal development are more serious than is usually the case with coronal synostosis; therefore, genetic testing is recommended when the more common diagnostic possibilities (involving mutations in *TWIST1*, *FGFR3*, *FGFR2*, and *TCF12*)^3,4^ have been excluded.

## Figures and Tables

**Table 1:** Phenotypic Features of Individuals with *ZIC1* Mutations

**Subject ID**	**Reference ID**	**Gender**	**Mutation (cDNA) and Alteration (Protein)**	**Cranial Sutures**	**Number of Major Craniofacial Procedures**	**Other Brain Abnormalities on CT/MRI Scanning**	**Strabismus/Ptosis**	**Learning Disability**	**Other Major Clinical Features**
1	4447	M	c.1163C>A (p.Ser388^∗^)	bicoronal synostosis	3	abnormal configuration of ventricles and corpus callosum	–	moderate-severe	scoliosis, foreskin stricture
2	4098	F	c.1204G>T (p.Glu402^∗^)	bicoronal synostosis	0	agenesis of corpus callosum, dilated lateral ventricles	divergent strabismus	moderate	scoliosis
3	4133/5847	M	c.1204G>T (p.Glu402^∗^)^a^	bicoronal synostosis, bony defect of sagittal suture	2	normal on CT scan	–	mild	–
4	12D11570	M	c.1165C>T (p.Gln389^∗^)	bicoronal synostosis, partial R lambdoid synostosis, bony defect of metopic and sagittal sutures	1	mildly enlarged lateral ventricles, shortened corpus callosum, hypoplastic pons, enlarged foramen magnum	strabismus sursoadductorius	moderate-severe	–
5:II.2	12D15615	F	c.1198G>C (p.Gly400Arg)	brachycephaly, delayed closure anterior fontanelle	0	atrophy of the rostral part of the cerebellum and pons	–	mild	–
5:II.4	08D1850	F	c.1198G>C (p.Gly400Arg)	plagiocephaly	0	reduced dorsum of pons, minor posterior fossa abnormalities	strabismus correction, ptosis L eye	below average	–
5:III.1	14D6457	M	c.1198G>C (p.Gly400Arg)	delayed closure anterior fontanelle (4 years)	0	DWM	–	mild	50 dB sensorineural hearing loss R; spina bifida occulta; LHRH deficiency
5:III.3	12D15613	M	c.1198G>C (p.Gly400Arg)	bicoronal synostosis, patent metopic, bilateral bony defect of lambdoid sutures	0	normal on CT scan	bilateral convergent strabismus + ptosis	mild	–
5:III.6	10D5797	M	c.1198G>C (p.Gly400Arg)	bicoronal synostosis, bilateral parietal foramina	1	reduced dorsum of pons, minor posterior fossa abnormalities	bilateral divergent strabismus + ptosis R eye	below average	–

a Mutation present in mosaic state.

## Web Resources

The URLs for data presented herein are as follows:

1000 Genomes, http://browser.1000genomes.org
dbSNP, http://www.ncbi.nlm.nih.gov/projects/SNP/
ExAC Browser, http://exac.broadinstitute.org/
NHLBI Exome Sequencing Project (ESP) Exome Variant Server, http://evs.gs.washington.edu/EVS/
OMIM, http://www.omim.org/
RefSeq, http://www.ncbi.nlm.nih.gov/RefSeq

## Supplemental Data

Document S1. Tables S1 and S2, Supplemental Case Reports, and WGS500 Member List

Document S2. Article plus Supplemental Data
