# Supplementary material for: Gain-of-Function Mutations in ZIC1 Are Associated with Coronal Craniosynostosis and Learning Disability
Source: Am J Hum Genet. 2015 Sep 3;97(3):378–88. doi: 10.1016/j.ajhg.2015.07.007 (PMC4564895; doi:10.1016/j.ajhg.2015.07.007)
Supplement: Document S1. Tables S1 and S2, Supplemental Case Reports, and WGS500 Member List [file mmc1.pdf]

The American Journal of Human Genetics

Supplemental Data

Gain-of-Function Mutations in *ZIC1* Are Associated with Coronal Craniosynostosis and Learning Disability

Stephen R.F. Twigg, Jennifer Forecki, Jacqueline A.C. Goos, Ivy C.A. Richardson, A. Jeannette M. Hoogeboom, Ans M.W. Van den Ouweland, Sigrid M.A. Swagemakers, Maarten H. Lequin, Daniel Van Antwerp, Simon J. McGowan, Isabelle Westbury, Kerry A. Miller, Steven A. Wall, WGS500 Consortium, Peter J. van der Spek, Irene M.J. Mathijssen, Erwin Pauws, Christa S. Merzdorf, and Andrew O.M. Wilkie

Dominant/de novo variants							
	chr	position	from	to	gene	type	comment
1	3	147131157	C	A	*ZIC1*	Stop	*de novo*
2	9	140777194	A	AGCGGCT	*CACNA1B)*	splicing	maternal
3	13	21746477	C	CGTGTA	*SKA3*	splicing	artefact/present in multiple samples
4	1	39879157	A	C	*KIAA0754*	nonsynonymous	artefact/present in multiple samples
5	1	145343385	C	A	*NBPF10*	nonsynonymous	present in multiple samples
6	1	144823868	T	G	*NBPF9*	nonsynonymous	present in multiple samples
7	1	144815968	G	A	*NBPF9*	nonsynonymous	present in multiple samples
8	1	7890026	A	G	*PER3*	nonsynonymous	artefact/present in multiple samples
9	4	88537261	A	T	*DSPP*	nonsynonymous	maternal
10	4	85998	T	A	*ZNF595*	nonsynonymous	present in multiple samples
11	6	168377071	G	A	*HGC6.3*	nonsynonymous	paternal
12	6	168377029	G	A	*HGC6.3*	nonsynonymous	paternal
13	8	144940209	T	C	*EPPK1*	nonsynonymous	artefact
14	12	50745703	T	G	*FAM186A*	nonsynonymous	artefact
15	15	82637061	C	T	*GOLGA6L10*	nonsynonymous	artefact/present in multiple samples
16	15	20740611	C	T	*GOLGA6L6*	nonsynonymous	paternal
17	16	22545467	C	T	*LOC100132247*	nonsynonymous	artefact/present in multiple samples
18	16	88599705	G	C	*ZFPM1*	nonsynonymous	artefact/present in multiple samples
19	16	88599703	T	C	*ZFPM1*	nonsynonymous	artefact/present in multiple samples
20	17	18544392	T	C	*TBC1D28*	nonsynonymous	maternal
21	17	15620506	G	A	*ZNF286A*	nonsynonymous	maternal
22	19	53553339	C	G	*ERVV-2*	nonsynonymous	paternal
23	19	56104423	A	C	*FIZ1*	nonsynonymous	paternal
24	X	8434191	G	A	*VCX3B*	nonsynonymous	present in multiple samples
25	1	144615246	A	AAG	*NBPF9*	frameshift insertion	artefact/present in multiple samples
26	19	14070706	A	AGGTGGG CCCAGGG CGGGCAG	*DCAF15*	frameshift insertion/splicing	artefact/present in multiple samples
27	Y	21154527	CTGCGTGGG	C	*CD24*	frameshift deletion	artefact/present in multiple samples
28	1	248524966	A	AGCTCTA CTTAGT	*OR2T4*	nonframeshift insertion	artefact/present in multiple samples
29	4	88535832	A	ATAGCAG TGACAGC AGCAG	*DSPP*	nonframeshift insertion	artefact/present in multiple samples
30	6	34857302	G	GGGCGGC	*ANKS1A*	nonframeshift insertion	paternal
31	11	8414087	G	GCCAGAA	*STK33*	nonframeshift insertion	present in both parents

32	12	125478381	C	CCTG	*BRI3BP*	nonframeshift insertion	present in multiple samples
33	17	72889649	C	CGTAGGT TCCATGG GCTCCGT A	*FADS6*	nonframeshift insertion	artefact/present in multiple samples
34	1	26608853	GCCGGGACCGGG ACCGGGACTGGG GCCGGGA	G	*UBXN11*	nonframeshift deletion	paternal and other samples
35	5	60628153	GGGCGGC	G	*ZSWIM6*	nonframeshift deletion	paternal
36	6	45390486	AGGCGGCGGCGG CGGCTGC	A	*RUNX2*	nonframeshift deletion	paternal and other samples
37	11	117789312	CGGGCTGGAGATG CCT	C	*TMPRSS13*	nonframeshift deletion	paternal and other samples
38	16	29821419	TGGCGGC	T	*MAZ*	nonframeshift deletion	paternal
39	17	43319434	TCCG	T	*FMNL1*	nonframeshift deletion	present in multiple samples
**Recessive variants**							
1	2	99013251	G	A	*CNGA3*	nonsynonymous	Homozygous; p.(Val540Ile)
2	2	152376170	A	G	*NEB*	splicing	paternal
	2	152530992	A	C	*NEB*	nonsynonymous/splicing	maternal

Table S1. Whole genome sequence analysis

Screening

Amplicon	Primer sequence 5′→3′ (M13 tags in lowercase) Forward	Reverse	Product (bp)	Amplification conditions[a]
Ex1a F/R	gtaaaacgacggccagt GCCGGGGCTCGCCCCGAGCAGCCACG	agcggataacaatttcacacagga ATCTGCCCGTTGACCACGTTAG	555	65°C + DMSO
Ex1b F/R	gtaaaacgacggccagt TTCCCCGGGCTTCACGAGCAGGCTGC	agcggataacaatttcacacagga GATCCCACCGAGGCTGCGTTTGTGCGACC	698	65°C + DMSO
Ex2 F/R	gtaaaacgacggccagt TTTTAAGCTTGCAAAGTGCTAATCCTG	agcggataacaatttcacacagga CCAAGAGAGCTCCTGCCTCAAAG	385	65°C
Ex3 F/R	gtaaaacgacggccagt GGGCTCCAAGGGGTCCAGGAGGAAGGG	agcggataacaatttcacacagga GTGTATACGTGTGTGATCAGTCTCTTAAATAGGG	404	65°C

Primers and amplification conditions for Subject 1 cDNA[b]

cDNA Fragment	Primer sequence 5′→3′ Forward	Reverse	Product size (bp)	Amplification conditions[a]	Digest
Exon 2-3	GGGAGAAGCCCTTCAAGTGCGAGTTTGAGGG	CGCAGGGTTCTTTCAGTAATGTTGTGTATAC	446	+ DMSO	BfaI(+)

Multiplex ligation-dependent probe amplification (MLPA)[c]

Probe	Primer sequence 5′→3′ Forward	Reverse	Product size (bp)
Exon 1	gggttccctaagggttggaCATCTGCTTCTGGGAGGAGTGTCCGCGCGAGGGCAAGCCCTTCAA AGCCAAA	TACAAACTGGTTAACCACATCCGCGTGCACACGtctagattggatcttgctggcac	127
Exon 2	gggttccctaagggttggaCAGTGCGAGTTTGAGGGCTGTGACCGGCGCTTCGCTA	ACAGCAGCGACCGCAAGAAGCACATGCCtctagattggatcttgctggcac	107
Exon 3	gggttccctaagggttggaGCCAACTGTTTGTACTGAATGGCAAGAATGTTCTAGTAAATGTGT ACCAA	AATGTGAATTACTTTGTACGATTACAGTCTCCACGTCGACCTAACCCtctagattggatcttgctggcac	139

cDNA cloning and mutagenesis

	Primer sequence 5′→3′ Forward	Reverse	
cDNA PCR[d]	CGC*CTCGAG*CAGCCACGATGCTCCTGG	CGATGTTTTGT*TCTAGA*TTTAAACGTACCATTCG	1380 bp
S388*	GAATCCTCCT**A**GCAGGGCTCG	GTGGACCTTCATGTGTTTGC	
E402*	CTCTGGCTAC**T**AATCCTCCACGCCTCCC	CTGGCGGCCGGCGAAGGC	
S436*	CGGCCACAGT**TA**GCTCTCTTCCAATTTTAAC	GCTGTGTGGTGGACTGCG	
E299*	GCACACAGGG**T**AGAAGCCCTT	GTCCTTTTGTGGATCTTTAAATTCTC	
T414A	ATCGTGTCTCCCTCC**G**CAGACAACCCGACCACA	TGTGGTCGGGTTGTCTG**C**GGAGGGAGACACGAT	
G400R	CCGGCCGCCAGCTCTC**G**CTACGAATCCTCCACG	CGTGGAGGATTCGTAG**C**GAGAGCTGGCGGCCGG	

cDNA Ion Torrent PGM sequencing[e]

	Primer sequence 5′→3′ Forward	Reverse	
ZIC1 PGM f/r	CGCTCTTCCGATCTCTGcacgagcgacaagccctatctttgcaag	TGCTCTTCCGATCTGACtaaggagcttgtggtcgggttgtctgtg	200 bp
A-BC1Rdfw	CCATCTCATCCCTGCGTGTCTCCGACTCAGacgagtgcgtCGCTCTTCCG ATCTCTG	CCTCTCTATGGGCAGTCGGTGATTGCTCTTCCGATCTGAC	P1-Rdrev

Table S2. Primers and amplification conditions. [a]DNA was obtained from whole blood samples by phenol-chloroform extraction and was amplified in a total volume of 20 µl containing 15 mM TrisHCl (pH 8.0), 50 mM KCl, 2.5 mM $MgCl_2$, 100 µM each dNTP, 0.4 µM primers, and 0.5 units of FastStart Taq (Roche) with or without 10% DMSO. Cycling conditions consisted of an 8 min denaturation step at 94°C, followed by 35 cycles of 94°C for 30 s, 65°C for 30 s and 72°C for 30s, with a final extension at 72°C for 10 min. [b]Mutation confirmation was carried out by PCR using the above conditions and indicated primers, followed by restriction digest of 8 µl of PCR product. [c]Multiplex-ligation-dependent probe amplification was performed using synthetic oligonucleotide probes designed to *ZIC1* according to protocols available from MRC-Holland: http://www.mrc-holland.com/pages/indexpag.html. Fragments were analyzed by capillary electrophoresis using an ABI 3130 containing POP-7 polymer. Peaks were visualized using Gene Mapper v3.7 (Applied Biosystems). Common PCR primer annealing sequences are shown in lower case, hybridizing sequences are shown in upper case and the 3' probe sequence is 5' phosphorylated.[d]cDNA amplification was performed using pCR4-Topo-ZIC1 (ThermoFisher) as template and PCR conditions as described above except that the high fidelity FastStart Taq (Roche) was used. Bold letters within the primer sequences are the mutated bases. The amplification product was cloned into pcDNA3 using XhoI and XbaI (sites are in italics in the primer sequences). [e]Amplification to generate templates for PGM sequencing was performed on fibroblast cDNA (see Methods) using primers PGM F and PGM R (*ZIC1* specific sequences are shown in lowercase) and the conditions as above except that the cycle number was 30. The product was diluted 1 in 100 and a second amplification carried out to add the Ion Torrent PGM adapters P1 and A with conditions as above except that high fidelity FastStart Taq (Roche) was used and cycle number was reduced to 8. Lowercase bases in the A-BC1-Rdfw primer correspond to barcode sequence.

Case reports

Subject 1

This male was the first child born to a healthy unrelated couple, aged 38.9 years (father) and 28.5 years (mother) at the time of birth. The mother had four healthy children by a previous relationship. A brother born subsequently had metopic synostosis but this is presumed to be coincidental since he had normal development and was negative for the *ZIC1* mutation.

During the pregnancy, renal dilatation had been noted at 22 weeks' gestation; a fetal blood sample showed a normal karyotype. The proband was born at 37 weeks' gestation by Caesarean section owing to persistent transverse lie, weighed 2.98 kg and was noted to have an abnormal head shape at birth. On assessment aged 20 weeks, his occipito-frontal circumference (OFC) was 38.5 (-3.5 SD). He was noted to have severe brachycephaly, a high forehead, downslanting palpebral fissures and an open posterior fontanelle. The remainder of the examination was normal except for a transverse palmar crease on the right hand. The computed tomography (CT) head scan showed bilateral coronal synostosis and no identifiable cerebral abnormality. He underwent a fronto-orbital advancement and remodelling procedure at the age of 7 months. By the age of 8 months he was noted to have developmental delay, with age-equivalent development at 4-5 month level. On formal assessment at 29 months using Bayley Scales of Infant Development (2nd Ed), his motor development was equivalent to 17 months and mental development to 12 months. Hearing and vision assessments were normal, although he previously required insertion of grommets. At 2.5 years his intracranial pressure (ICP) was measured because of concerns about slow head growth and delayed development, this was markedly increased (baseline 25-30 mm Hg, peaks up to 50 mm Hg) and he underwent a posterior release and remodelling procedure. By 3 years he was showing hyperactive behaviour, with sleep disturbance and head-banging. A repeat ICP measurement aged 4.6 years was again elevated with episodes of abnormal pressure in the 25-35 mm Hg range, and he underwent a posterior release and advancement procedure.

At the age of 5 years he was noted to have a stricture of his foreskin and was developing a scoliosis. By 8 years, the left sided thoraco-lumbar curve measured 50°, and he had extensive spina bifida occulta in his lower lumbar spine; he subsequently had spinal surgery at the age of 10 years. He started to develop intermittent outbursts of aggressive behaviour, but several measurements of ICP were normal. A magnetic resonance imaging (MRI) brain scan showed abnormal configuration of the ventricles and corpus callosum. His developmental progress was slow and he attended a school for children with special educational needs. Genetic testing for causes of learning disability including repeat karyotype, telomere screen, *FRAX* and *PW71B* methylation (for Prader-Willi/Angelman syndrome) were normal. Testing for craniosynostosis mutations in the research laboratory (*FGFR2*, *FGFR3*, *TWIST1*) was normal. On assessment at the age of 13 years, he continued to have violent outbursts and poor sleeping routine. He had no language and only limited communication using signs, but his gross motor skills and comprehension were less severely delayed. He showed autistic features, being dependent on routines and focused on individual tasks.

Subject 2

This female was the only child of healthy unrelated parents aged 34.4 years (father) and 31.7 years (mother). She was born at term by forceps delivery following a normal pregnancy and weighed 3.23 kg. Although well at birth she fed poorly and was referred at the age of 4 months for assessment of an unusual head shape and dysmorphic appearance with "almond-shaped eyes" and down-slanted palpebral fissures, when a skull radiograph showed craniosynostosis. A clinical geneticist noted a high forehead, large open anterior fontanelle, flat occiput, maxillary hypoplasia and normal extremities, and suggested a diagnosis of Crouzon syndrome. Her development was moderately delayed; she walked unsupported at 2 years and had a vocabulary of 30 words at 2.8 years. She attended special schools for children with moderate to severe learning disability. She had surgery for divergent strabismus aged 3 years. CT head scan at 9 years showed dilated lateral ventricles and agenesis of the corpus callosum. At the age of 13 years she was noted to have a kyphoscoliosis. Plain radiographs showed multiple abnormalities of the thoracic spine and ribs and spondylolisthesis at L5/S1. MRI scan aged 17 years confirmed the previous findings and demonstrated a retroverted odontoid peg impinging on the cranio-cervical junction. There was no deterioration on orthopaedic follow-up to the age of 21 years.

She was referred for craniofacial assessment at the age of 23 years. Her head circumference was 48.5 cm (-5 SD) and height 149 cm (-2.2 SD). She was noted to be markedly brachycephalic with a high, flat brow and low frontal hairline. The remainder of the examination was normal and did not reveal diagnostic features of any craniosynostosis syndrome. Genetic testing (*FGFR1*, *FGFR2*, *FGFR3*, *TWIST1*) was negative and the karyotype was normal. CT scanning was consistent with bicoronal synostosis and demonstrated dilatation of the trigone and temporal regions of the ventricles. No surgical intervention was undertaken in view of her stable state. Currently aged 36 years, she lives in supervised residential accommodation, is able to communicate in short sentences and wash, dress and feed herself, but requires help with cooking.

Subject 3

This boy was the first child born to healthy unrelated parents after a normal pregnancy and weighed 3.34 kg (-0.1 SD). He was noted to have brachycephaly shortly after birth and was referred for craniofacial assessment aged 6 weeks. At 19 weeks his OFC was 39.0 cm (-2.8 SD) and cephalic index was 1.24. He had a low frontal hairline and normal extremities; on clinical genetic assessment his appearance was considered consistent with either Muenke or Saethre-Chotzen syndrome, but genetic testing of *FGFR3* and *TWIST1* was normal. The CT scan at the age of 5 months showed bicoronal synostosis, a patent metopic suture, a large wormian bone in the position of the anterior fontanelle, and an ossification defect in the sagittal suture. He developed progressive turricephaly and underwent a posterior distraction procedure aged 8 months followed by fronto-orbital advancement and remodelling aged 2.4 years.

He had mild developmental delay with speech dysfluency, and occasional tantrums with changes in routine. Tests of vision and hearing were normal. At the age of 8 years he was attending a normal school but was receiving 1:1 educational support because of delayed learning. Array comparative genomic hybridisation was normal except for a 140 kb deletion

on chromosome 17 that was inherited from the mother and therefore presumed to be coincidental to the phenotype.

Subject 4

This boy was the fourth child of healthy unrelated parents aged 41 years (father) and 36 years (mother); an older sibling had trisomy 18. The proband was born at 38 weeks' gestation, weighing 4.27 kg. An altered skull shape was immediately apparent; on assessment by the plastic surgery department at the age of 2.5 months, this was clinically consistent with bilateral coronal synostosis and microcephaly (OFC 36 cm; -2.1 SD), but without significant exorbitism, ptosis or malformations of the extremities. 3D CT scan showed bilateral coronal synostosis and a partial right lambdoid synostosis; the metopic and mid-part of the sagittal suture were widely patent. A clinical diagnosis of Muenke or Saethre-Chotzen syndrome was considered, but appropriate genetic testing was negative. Fronto-orbital and supraorbital rim remodeling, and osteotomy of the right lambdoid suture were performed at the age of 7 months.

On reassessment at the age of one year, delayed development was apparent. An MRI brain scan at 16 months showed a short corpus callosum, mildly enlarged lateral ventricles, a peaked tentorium cerebelli, enlarged foramen magnum with hypoplastic pons and cerebellum with prominent cerebellar folia and signal void near the cervical cord. The left transverse and sigmoid sinuses, together with the jugular foramen were enlarged, whereas on the right the transverse and sigmoid sinuses were underdeveloped. Ophthalmological assessment at the age of 18 months showed esotropia of the right eye, latent nystagmus, strabismus sursoadductorius with V motility and bilateral granular pigmented retinae. Metabolic testing was normal. Adenotonsillectomy was performed at the age of 2 years because of recurrent tonsillitis and otitis media.

Development continued to be delayed and was formally assessed at the age of 34 months. Motor development was at 15 months equivalent, but no formal testing of mental development or speech was possible. His back was hyperpigmented and pedes plano valgi were present. At 4 years, speech and gait were noted to be ataxic and autistic spectrum disorder and attention deficit hyperactivity disorder were diagnosed. Optic nerve hypoplasia was noted, worse in the right eye in which vision was reduced to 30% of normal; the left eye was myopic (-2.25 diopters).

Subject 5:III.3 (proband)

This male was the second child born at 38 weeks' gestation to unrelated parents. His grandfather (I.1), mother (II.2) and half-brother (III.1) had a similar head shape (see below). He weighed 2.94 kg and was well except for neonatal jaundice. At 30 months a brachycephalic head shape was noted with OFC of 46 cm (-2.5 SD), associated with patent posterior fontanelle (3.3 cm), an asymmetrical face, beaked nose, a high palate, and downslant and ptosis of the eyes. No hearing deficits or malformations of the upper or lower extremities were present. A clinical diagnosis of Saethre-Chotzen syndrome was suggested.

His development was generally delayed; he started walking at the age of 2.5 years and had delayed speech. He was generally hypotonic with joint laxity. An electroencephalogram

(EEG) showed an irregular and diffuse disordered background pattern with accentuated frontal dysfunction. He had a bilateral convergent strabismus.

He was referred for plastic surgical assessment at the age of 3.5 years. The craniofacial features were as noted previously. Vision was normal with glasses. Skull radiographs showed bicoronal synostosis with increased thumbprinting of the frontal bones. CT head scanning demonstrated in addition a patent metopic suture, bilateral bony defect of the lambdoid suture, and normal ventricles and posterior fossa. No surgery was performed because of his age.

His most recent review was at the age of 19 years. He still complained of headaches. He had a mildly delayed development and lived under social supervision. His communication was normal. Mild flattening of the left supra-orbital region, mild asymmetry of the skull, and downslant and exorbitism of the eyes were present.

Subject 5:III.1

The older half-brother of the proband was born at 40 weeks' gestation with a weight of 4.00 kg and a length of 52 cm. He was well during infancy but was clumsy with mildly delayed developmental milestones (walked at 3 years, rode a bicycle at 6 years, swimming certificate at 12 years). He had a large anterior fontanelle that did not close until 4 years of age. Attention deficit disorder was diagnosed.

At the age of 8 years he was investigated for suspected hearing loss. He had persisting central hypotonia, was dysarthric, mildly dysphasic and dyspraxic, and was unable to heel-toe walk. Hyperpigmentation was noted over his back. He was found to have a 50 dB left-sided sensorineural hearing loss; auditory evoked potentials suggested a sensorineural cause. CT head scan showed cystic dilatation of the 4th ventricle communicating with the cisterna magna, and hypoplasia of the cerebellar vermis. The third and lateral ventricles and the foramina of Luschka and Magendi were normal. These features were consistent with a variant of Dandy-Walker malformation. Visual evoked potentials were normal and a radio-iodinated serum albumin brain scan suggested obstruction of drainage of the left lateral ventricle. Radiology of the spine showed hypolordosis of the cervical spine and spina bifida occulta with absent arches of the L4, L5 and S1 vertebrae.

During the latter part of childhood his symptoms persisted. At the age of 15 years LHRH deficiency was diagnosed because of pubertal delay and he was started on pulsatile LHRH therapy. On further assessment aged 16 years, his OFC was 53 cm (-1.96 SD) and hyperpigmentation of his back was present. An MRI scan confirmed the earlier CT findings and showed atrophy of the cerebellum, the brachium pontis and temporo-basal lobes with widened sulci.

Subject 5:II.2

Little detailed information is available on the mother of III-1 and III-3. She reported that as a child she had an enlarged late-closing fontanel, and had learning disability requiring her to repeat classes in both primary and secondary school. She suffered from vertigo and had a clumsy tandem gait. MRI scan showed atrophy of the rostral part of the cerebellum and pons, similar to, but less severe than, III-1.

The family was investigated for a suspected autosomal dominant cerebellar atrophy, but relevant genetic testing (*SCA1*, *SCA2*, *SCA3* and *SCA6*) was negative in both 5:II.2 and 5:III.1.

Subject 5:III.6

The proband's male cousin, the 3rd child in the sibship (the two older siblings were healthy) was born at 37 weeks' gestation after an uneventful pregnancy, weighing 3.35 kg. He was referred for a plastic surgery opinion at one month of age because of concerns about his head size. He was microcephalic (OFC 38.5cm at 3.5 months; -1.9 SD) and noted to have brachycephaly, hypoplastic supraorbital ridges, hypertelorism, mild ptosis of the right eye, downslanting palpebral fissures, divergent strabismus, a high narrow palate, normal ears and symmetrical defects palpable over the occipito-parietal region. Additional features noted were axial hypotonia (but with increased muscle tone of the upper extremities), mild tapering of the fingers, clinodactyly of the 4th and 5th toes and a sacral dimple. A clinical diagnosis of Saethre-Chotzen syndrome was suggested. 3D CT scanning at the age of 2 months showed bilateral coronal synostosis with bilateral large parietal foramina. Ultrasound of the brain was normal. A fronto-orbital advancement was performed at the age of 11 months. The divergent strabismus persisted and posterior tenotomies were performed at the age of 3 years and 5 months.

At the age of 13 years an MRI brain scan was performed. The fourth ventricle and both superior cerebellar peduncles were mildly enlarged, with a prominent great cerebral vein and inferior sagittal sinus. The posterior fossa was small with a peaked tentorium cerebelli, short and steep straight sinus with mild hypoplasia of the dorsal part of the pons and inferior part of the cerebellar vermis. There was no Chiari or Dandy-Walker malformation.

His schooling history is not documented. Formal psychological testing was performed at the age of 16 years. The WISCIII scores were full scale IQ 89, verbal IQ 96, performance IQ 84. The Child Behavior Checklist 6-18, Social Communication Questionnaire, and Children's Communication Checklist-2 NL were normal. The OFC was 55cm (0 SD). He had small teeth and wore orthodontic braces.

Subject 5:II.4

This individual is the sister of II.2 and mother of III.6. As a child she had a strabismus correction, but no other surgery. She had mildly delayed development with attention deficit, aberrant fine motor skills and clumsiness, but attended a normal school. Her hearing and balance were normal. Currently aged 53 years, she complains of persistent severe headaches, forgetfulness and dental problems. On examination her height was 1.62 m (-0.3 SD), she had brachymicrocephaly (OFC 51cm, -2.9 SD), vertical orbital dystopia, ptosis of the left upper eyelid, midface hypoplasia, nasal deviation, and facial and occlusal asymmetry with narrow maxilla, crowding of the upper front teeth, absence of the lower right first molar and upper right canine, and prominent crura of both ears. The hands and feet were normal. MRI brain scan showed asymmetry of the skull base and head shape, an enlarged foramen magnum, a peaked tentorium cerebelli, mildly hypoplastic inferior part of the cerebellar vermis and possibly a small defect of the hiatus tentorium cerebelli.

Subject 5:I.1

The deceased father of II.2 and II.4 (I.1) had a high forehead, facial asymmetry and hearing deficits, suggesting that the *ZIC1* mutation present in his two daughters had been transmitted from him.

WGS500: names and affiliations of authors

Steering Committee: Peter Donnelly (Chair)[1], John Bell[2], David Bentley[3], Gil McVean[1], Peter Ratcliffe[1], Jenny Taylor[1,4], Andrew Wilkie[4,5]

Operations Committee: Peter Donnelly (Chair)[1], John Broxholme[1], David Buck[1], Jean-Baptiste Cazier[1], Richard Cornall[1], Lorna Gregory[1], Julian Knight[1], Gerton Lunter[1], Gil McVean[1], Jenny Taylor[1,4], Ian Tomlinson[1,4], Andrew Wilkie[4,5]

Sequencing & Experimental Follow up: David Buck (Lead)[1], Christopher Allan[1], Moustafa Attar[1], Angie Green[1], Lorna Gregory[1], Sean Humphray[3], Zoya Kingsbury[3], Sarah Lamble[1], Lorne Lonie[1], Alistair Pagnamenta[1], Paolo Piazza[1], Guadelupe Polanco[1], Amy Trebes[1]

Data Analysis: Gil McVean[1] (Lead), Peter Donnelly[1], Jean-Baptiste Cazier[1], John Broxholme[1], Richard Copley[1], Simon Fiddy[1], Russell Grocock[3], Edouard Hatton[1], Chris Holmes[1], Linda Hughes[1], Peter Humburg[1], Alexander Kanapin[1], Stefano Lise[1], Gerton Lunter[1], Hilary Martin[1], Lisa Murray[3], Davis McCarthy[1], Andy Rimmer[1], Natasha Sahgal[1], Ben Wright[1], Chris Yau[6]

[1]The Wellcome Trust Centre for Human Genetics, Roosevelt Drive, Oxford, OX3 7BN, UK

[2]Office of the Regius Professor of Medicine, Richard Doll Building, Roosevelt Drive, Oxford, OX3 7LF, UK

[3]Illumina Cambridge Ltd., Chesterford Research Park, Little Chesterford, Essex, CB10 1XL, UK

[4]NIHR Oxford Biomedical Research Centre, Oxford, UK

[5]Weatherall Institute of Molecular Medicine, John Radcliffe Hospital, Headington, Oxford OX3 9DS, UK

[6]Imperial College London, South Kensington Campus, London, SW7 2AZ, UK
