# Supplementary material for: Gain-of-Function Mutations in ZIC1 Are Associated with Coronal Craniosynostosis and Learning Disability
Source: Am J Hum Genet. 2015 Sep 3;97(3):378–88. doi: 10.1016/j.ajhg.2015.07.007 (PMC4564895; doi:10.1016/j.ajhg.2015.07.007)
Supplement: Document S2. Article plus Supplemental Data [file mmc2.pdf]

Gain-of-Function Mutations in *ZIC1* Are Associated with Coronal Craniosynostosis and Learning Disability

Stephen R.F. Twigg,[1] Jennifer Forecki,[2,10] Jacqueline A.C. Goos,[3,10] Ivy C.A. Richardson,[4,11] A. Jeannette M. Hoogeboom,[5] Ans M.W. van den Ouweland,[5] Sigrid M.A. Swagemakers,[6] Maarten H. Lequin,[7] Daniel Van Antwerp,[2] Simon J. McGowan,[8] Isabelle Westbury,[1] Kerry A. Miller,[1] Steven A. Wall,[9] WGS500 Consortium, Peter J. van der Spek,[6] Irene M.J. Mathijssen,[3] Erwin Pauws,[4] Christa S. Merzdorf,[2] and Andrew O.M. Wilkie[1,9,*]

Human *ZIC1* (zinc finger protein of cerebellum 1), one of five homologs of the *Drosophila* pair-rule gene *odd-paired*, encodes a transcription factor previously implicated in vertebrate brain development. Heterozygous deletions of *ZIC1* and its nearby paralog *ZIC4* on chromosome 3q25.1 are associated with Dandy-Walker malformation of the cerebellum, and loss of the orthologous *Zic1* gene in the mouse causes cerebellar hypoplasia and vertebral defects. We describe individuals from five families with heterozygous mutations located in the final (third) exon of *ZIC1* (encoding four nonsense and one missense change) who have a distinct phenotype in which severe craniosynostosis, specifically involving the coronal sutures, and variable learning disability are the most characteristic features. The location of the nonsense mutations predicts escape of mutant *ZIC1* transcripts from nonsense-mediated decay, which was confirmed in a cell line from an affected individual. Both nonsense and missense mutations are associated with altered and/or enhanced expression of a target gene, *engrailed-2*, in a *Xenopus* embryo assay. Analysis of mouse embryos revealed a localized domain of *Zic1* expression at embryonic days 11.5–12.5 in a region overlapping the supraorbital regulatory center, which patterns the coronal suture. We conclude that the human mutations uncover a previously unsuspected role for Zic1 in early cranial suture development, potentially by regulating *engrailed 1*, which was previously shown to be critical for positioning of the murine coronal suture. The diagnosis of a *ZIC1* mutation has significant implications for prognosis and we recommend genetic testing when common causes of coronal synostosis have been excluded.

Introduction

Among the varied causes of craniosynostosis (premature fusion of one or more sutures of the skull vault), a monogenic etiology is most commonly identified in individuals with fusion of the coronal sutures, the major pair of transverse sutures crossing the vertex of the skull.[1] Coronal synostosis, which can be present bilaterally (bicoronal) or unilaterally (unicoronal), affects approximately 1 in 10,000 children[2] and is the type most commonly associated with an identifiable syndrome. Common monogenic disorders that characteristically present with coronal synostosis are Muenke (MIM: 602849) and Apert (MIM: 101200) syndromes, caused by localized gain-of-function mutations encoded by *FGFR3* (MIM: 134934) and *FGFR2* (MIM: 176943), respectively; Saethre-Chotzen syndrome (MIM: 101400) (*TWIST1* [MIM: 601622] haploinsufficiency); *TCF12*-related craniosynostosis (MIM: 600480 and 615314) (also a haploinsufficiency); and craniofrontonasal syndrome (MIM: 304110) (cellular interference involving variants in the X-linked *EFNB1* gene [MIM: 300035]).[3,4]

Even in the absence of an obvious syndromic diagnosis, a specific mutation can be identified in about 60% of individuals with bicoronal and 30% with unicoronal synostosis.[1,4] The high monogenic load in coronal synostosis can be accounted for by the specific developmental origin of the coronal suture, which lies at an embryonic tissue boundary between neural-crest-derived frontal bone and mesoderm-derived parietal bone.[5,6] Based on analysis of mouse models,[7] coronal synostosis is frequently caused by disruption in the maintenance of the population of stem cells within the suture during early development (typically, embryonic days [E]12.5–14.5), caused, for example, by abnormalities in migration of neural crest cells[8] or abnormal paracrine signaling through fibroblast growth factor receptors.[9,10]

An alternative possibility is that coronal synostosis could be caused by a primary failure of the suture to develop. Lineage tracing demonstrates that the cells of the future

[1]Clinical Genetics Group, Weatherall Institute of Molecular Medicine, University of Oxford, John Radcliffe Hospital, Headington, Oxford OX3 9DS, UK; [2]Department of Cell Biology and Neuroscience, 513 Leon Johnson Hall, Montana State University, Bozeman, MT 59717, USA; [3]Department of Plastic Surgery, Erasmus MC, University Medical Center Rotterdam, PO Box 2040, 3000 CA Rotterdam, the Netherlands; [4]Developmental Biology and Cancer Programme, UCL Institute of Child Health, 30 Guilford Street, London WC1N 1EH, UK; [5]Department of Clinical Genetics, Erasmus MC, University Medical Center Rotterdam, PO Box 2040, 3000 CA Rotterdam, the Netherlands; [6]Department of Bioinformatics, Erasmus MC, University Medical Center Rotterdam, PO Box 2040, 3000 CA Rotterdam, the Netherlands; [7]Department of Pediatric Radiology, Erasmus MC, University Medical Center Rotterdam, PO Box 2040, 3000 CA Rotterdam, the Netherlands; [8]Computational Biology Research Group, Weatherall Institute of Molecular Medicine, University of Oxford, John Radcliffe Hospital, Headington, Oxford OX3 9DS, UK; [9]Craniofacial Unit, Department of Plastic and Reconstructive Surgery, Oxford University Hospitals NHS Trust, John Radcliffe Hospital, Oxford OX3 9DU, UK
[10]These authors contributed equally to this work
[11]Deceased
*Correspondence: andrew.wilkie@imm.ox.ac.uk
http://dx.doi.org/10.1016/j.ajhg.2015.07.007. ©2015 The Authors
This is an open access article under the CC BY license (http://creativecommons.org/licenses/by/4.0/).

coronal suture originate from paraxial cephalic mesoderm at E7.5 and migrate laterally to locate above the developing eye.[11] This region constitutes the supraorbital regulatory center and during E11.5–E13.5, cells from this zone migrate apically to form and populate the coronal suture.[6,11,12] One of the genes characteristically expressed by these cells is *engrailed 1* (*En1*), a homolog of the *Drosophila engrailed* segment polarity gene. Mice with homozygous loss of *En1* function have generalized calvarial bone hypoplasia and persistent widening of the sutural gaps, which is associated with a posterior shift in the boundary between cells of neural crest and mesodermal origin.[11,13] An orthologous mutation has not yet been described in humans.

Here, we report an additional genetic etiology for coronal synostosis, caused by heterozygous variants in the final exon of *ZIC1* (zinc finger protein of cerebellum 1 [MIM: 600470]), identified in four simplex case subjects and a three-generation pedigree. *ZIC1*, located on chromosome 3q25.1, belongs to a family of five genes encoding Zn-finger transcription factors, which are arranged as one unpaired and two paired paralogs in the human and mouse genomes;[14] *ZIC* genes are homologous to the *Drosophila* pair-rule gene *odd-paired*, which is required for activation of embryonic *engrailed* expression.[15] Vertebrate ZICs have important roles in multiple developmental processes, including neurogenesis, left-right axis formation, myogenesis, and skeletal patterning.[16,17] Heterozygous complete deletions of *ZIC1* were previously associated with Dandy-Walker malformation (DWM; hypoplasia and upward rotation of the cerebellar vermis and cystic dilatation of the fourth ventricle [MIM: 220200]);[18] we now show that mutations affecting the highly conserved C terminus of the protein, which are likely to be associated with a gain of function, lead to a distinct phenotype of coronal suture fusion and learning disability. In addition to its previously established importance for neurogenesis,[19,20] this work shows that *ZIC1* is required for normal coronal suture development. We find that murine *Zic1* is expressed in the supraorbital regulatory center, suggesting that this gene acts at a very early stage of coronal suture development,[7] potentially (reflecting a similar epistatic relationship to that in *Drosophila*) by regulating *En1*.

Subjects and Methods

Subjects

The clinical studies were approved by Oxfordshire Research Ethics Committee B (reference C02.143), London Riverside Research Ethics Committee (reference 09/H0706/20), and the Medical Ethical Committee of the Erasmus University Medical Center Rotterdam (MEC-2012-140 and MEC-2013-547). Written informed consent to obtain samples for genetics research was obtained from each child's parent or guardian. Venous blood was used for DNA extraction and fibroblast cultures were established from skin biopsies taken from scalp incisions during surgical intervention. Intracranial pressures in subject 1 were documented by 24–48 hr direct recording with an intraparenchymal Codman Microsensor.[21] The screening panel comprised samples from 307 individuals with syndromic or non-syndromic craniosynostosis. All DNA samples were previously tested for mutation hotspots in *FGFR2*, *FGFR3*, *TWIST1*, and *TCF12*.[3,4] Significant chromosome aneuploidy in individuals with *ZIC1* mutations was excluded by karyotyping and/or array comparative genomic hybridization. Where necessary, correct biological relationships were confirmed by segregation analysis of a panel of 13 microsatellites (*D1S2868*, *D3S1311*, *D4S403*, *D5S2027*, *D6S1610*, *D7S519*, *D9S158*, *D10S548*, *D11S898*, *D13S1265*, *D14S280*, *D16S415*, and *D18S474*).

Whole Genome/Exome Sequencing and Mutation Screening of *ZIC1*

Whole genome sequencing (WGS) of the male proband subject 1 and his parents was performed as part of the WGS500 clinical genome sequencing initiative.[22] In brief, 3–5 μg DNA was used to prepare libraries for 100 bp paired-end sequencing to generate a mean coverage of 30× using the Illumina HiSeq2000 platform. Sequence reads were mapped to the human reference GRCh37d5 using Stampy (v1.0.12–1.0.22) and variants called with Platypus (v.0.2.4).[23,24] To identify de novo mutations, we prioritized variants within coding regions that were called as absent in both parents and in dbSNP135, generating a list of 203 variants in 177 genes, of which 39 were classified as protein altering. Visualization of the trio read alignments revealed a single bona fide change in *ZIC1* (12 of 27 reads), which was absent in both the paternal (19 reads) and maternal (31 reads) samples; the other 38 variants were either in fact present in one of the parents or were artifactual (Table S1). Possible recessive inheritance was analyzed with an in-house perl script to list homozygous, compound heterozygous, and hemizygous X chromosomal variants in subject 1, with a frequency cut-off of 0.003 in either 1000 Genomes or Exome Variant Server; variants in two genes fitted the criteria (Table S1). Whole genome sequencing of genomic DNA from four subjects in family 5 (affected: 5:II.2, 5:III.3, 5:III.6; unaffected: 5:II.3) was performed by BGI Complete Genomics.[25,26] Filtering based on a list of genes mutated in craniosynostosis identified a predicted missense substitution encoded by *ZIC1*, present only in the three affected individuals. Exome sequencing of subject 3 was performed on genomic DNA (extracted from whole blood) using an Agilent SureSelect Human All Exon Kit (v.5; 50 Mb) on the Illumina HiSeq2000 platform. Reads were mapped to hg19 with Novoalign (Novocraft Technologies) and variants called with SAMtools and annotated by ANNOVAR.

To investigate further the significance of the *ZIC1* mutations, primers were designed for amplification of genomic DNA (GenBank: NT_005612.17) and cDNA (GenBank: NM_003412.3), for multiplex ligation-dependent probe amplification (MLPA) analysis, and for deep sequencing (Table S2, which provides details of experimental conditions). Variant screening of all three exons of *ZIC1* was performed by dideoxy sequencing on PCR amplification products from genomic DNA by BigDye Terminator v3.1 (Applied Biosystems). Copy-number variation was analyzed by MLPA using probes to each exon, according to the manufacturer's instructions (MRC Holland). RNA was extracted from fibroblasts (Trizol, Invitrogen), cDNA synthesized with RevertAid first strand cDNA kit (Thermo Scientific), and the samples analyzed by agarose gel electrophoresis after digestion with BfaI. To quantify the proportions of wild-type to mutant allele in cDNA, an amplification product spanning exons 2–3 was used as a template for PCR to add Ion Torrent P1 and A adapters, and the resulting product

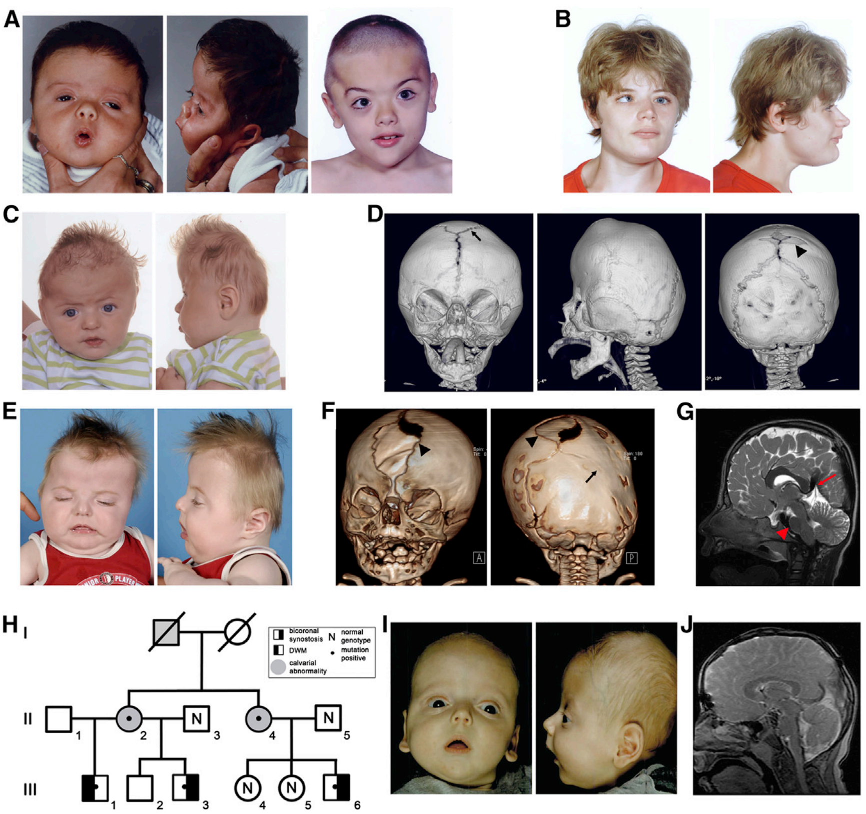

Figure 1. Clinical and Radiological Phenotype of Individuals with *ZIC1* Mutations
(A) Subject 1, age 7 weeks (left) and 8 years (right).
(B) Subject 2, age 23 years.
(C) Subject 3, age 5 months.
(D) CT head scan of subject 3 (age 5 months) showing bicoronal synostosis, a large wormian bone (arrow) in the position of the anterior fontanelle, and an ossification defect in the sagittal suture (arrowhead).
(E) Subject 4, age 7 months.
(F) CT head scan of subject 4 (age 2.5 months). Note asymmetric skull shape associated with bilateral coronal and right lambdoid suture fusion (arrow). Sections of the metopic and sagittal sutures remain widely patent (arrowheads).
(G) MRI brain scan (T2 image) of subject 4, age 16 months. Note short, broad corpus callosum, peaked tentorium cerebelli (arrow), and hypoplasia of the pons (arrowhead), cerebellar vermis, and cerebellar hemispheres.
(H) Pedigree of family 5.
(I) Subject 5:III.6, age 2 months.
(J) MRI brain scan (T2 image) of subject 5:III.6, age 13 years. Abnormal features show a similar pattern to those present in subject 4.

was purified with AMPure beads (Beckman Coulter). Emulsion PCR and enrichment were performed with the Ion PGM Template OT2 200 Kit (Life Technologies) according to the manufacturer's instructions and sequencing of enriched templates performed on the Ion Torrent PGM (Life Technologies) for 125 cycles with the Ion PGM Sequencing 200 kit v2. Data were processed with Ion Torrent platform-specific pipeline software v.4.2.1.

Xenopus Assays

All experiments using *Xenopus* were approved by the Institutional Animal Care and Use Committee of Montana State University. *Xenopus* full-length *zic1*[27] and *zic1ΔC*[28] cDNA constructs were described previously (*zic1ΔC* was originally termed *oplΔC*). The *zic1ΔC2* construct was made by PCR amplification of the portion of *zic1* cDNA encoding the N terminus and zinc finger domains, including four amino acids of the C-terminal region, followed by cloning into the EcoR1 and Xba1 sites of pCS2+ATG.[27] The human *ZIC1* cDNA pCR4-Topo-ZIC1 (ThermoFisher) was subcloned into pcDNA3 and six different nucleotide substitutions—c.895G>T (p.Glu299*), c.1163C>A (p.Ser388*), c.1198G>C (p.Gly400Arg), c.1204G>T (p.Glu402*), c.1240A>G (p.Thr414Ala), and c.1309_1310GC>TA (p.Ala437*)—were introduced by PCR mutagenesis using the primer sequences and experimental conditions provided in Table S2. The human *ZIC1* constructs were subsequently digested with EcoR1 and Xba1 and ligated into the pCS2+ plasmid. Capped sense RNAs for microinjection were synthesized from the *Xenopus* and human pCS2+ constructs by SP6 transcription of NarI linearized plasmids. *Xenopus laevis* eggs were collected and fertilized as previously described[28] and embryos were staged according to Nieuwkoop and Faber.[29] Embryos at the two-cell stage were injected into a single cell with 200 pg sense RNA synthesized from cDNA constructs, together with 25 pg *lacZ* RNA as tracer. After β-galactosidase staining,[30] wild-type embryos were bleached by exposing the embryos to fluorescent light in hybridization buffer containing 1% H_2O_2. Expression of *en-2* was determined in neurula stage 15–17 albino and wild-type embryos by in situ hybridization[31] with digoxygenin-labeled antisense RNA *en-2* probe as described.[32] An anti-digoxygenin alkaline phosphatase-conjugated antibody (Roche) and the alkaline phosphatase substrate NBT/BCIP (Fisher Scientific) were used for color detection. Embryos were scored double-blind to determine changes in *en-2* expression in comparison to the uninjected side. Results using wild-type and mutant constructs were compared by Fisher's exact tests with Bonferroni correction for multiple comparisons ($n = 9$).

RNA In Situ Hybridization of Mouse Embryos

Experimental procedures were performed in accordance with UK Animals (Scientific Procedures) Act, 1986 (PPL 70/7194). For whole-mount embryo in situ hybridization, embryos were dissected, fixed overnight in 4% paraformaldehyde in phosphate-buffered saline, and dehydrated through graded methanol solutions. Non-radioactive RNA in situ hybridization was performed as described[33] before vibratome sectioning. RNA probes for *Zic1*[34] and *En1*[35] were digoxygenin labeled with the In Vitro Transcription kit (Roche Applied Science) followed by anti-digoxygenin-AP antibody (1:1,000) (Roche Applied Science) and NBT/BCIP (Sigma) staining to detect the hybridization signals.

Results

Identification of *ZIC1* Mutations

The proband (subject 1) presented at birth with severe brachycephaly (Figure 1A), which was shown by three-

dimensional computed tomographic reconstruction (3D-CT) to be caused by bicoronal synostosis. He required three major craniofacial surgical procedures (at the ages of 7 months, 2.4 years, and 4.8 years), the latter two because of raised intracranial pressure. In addition he had autistic traits and moderate-severe learning disability, features that are rarely associated with coronal synostosis. Genetic testing for mutations known to be associated with coronal synostosis was negative; his phenotype is summarized in Table 1 and more detailed descriptions of all subjects are provided in the Supplemental Data (Case Reports).

We undertook WGS of the parent-child trio and analyzed the data for variants consistent with either (1) autosomal or X-linked recessive inheritance or (2) a new dominant mutation (Table S1). After filtering, variants in two genes were consistent with the recessive disease model, but these genes, *CNGA3* (MIM: 600053) and *NEB* (MIM: 161650), are associated with achromatopsia 2 (MIM: 216900) and nemaline myopathy 2 (MIM: 256030), respectively, disorders without a craniofacial phenotype, and thus were not considered further. The single dominant candidate was a heterozygous c.1163C>A mutation in *ZIC1* (GenBank: NM_003412.3), predicting the nonsense change p.Ser388*. This variant was absent in the parental samples and was confirmed by dideoxy sequencing (Figure 2A).

The significance of this de novo *ZIC1* mutation was initially uncertain. Contiguous heterozygous deletions of *ZIC1* and its adjacent paralog *ZIC4* were previously described in DWM,[18] although a few deletion cases have lacked this characteristic phenotype.[36] Review of the CT scan in subject 1 showed no brain malformation, and on a later magnetic resonance imaging (MRI) scan, only minor abnormalities of configuration of the ventricles and corpus callosum were evident (not shown).

Nonsense mutations of *ZIC1* have not previously been reported, but the location of the nucleotide substitution in the terminal (third) exon (Figure 2A) predicted that it would escape nonsense-mediated decay.[37] We digested *ZIC1* cDNA generated from scalp fibroblasts with the restriction enzyme BfaI, which cuts the mutant allele, and found that the expected mutant fragments were readily visualized (Figure 2B); we then used deep sequencing for accurate quantification and found that 7,175 of 13,088 reads (55%) represented mutant alleles, confirming escape from nonsense-mediated decay (not shown). A prematurely truncated translation product could be associated with dominant-negative or gain-of-function mechanism, distinct from the previously described deletions.[37] Support for a gain-of-function mechanism was provided by previously published work on the *Xenopus zic1* ortholog, formerly known as *opl* (*odd-paired*-like), in which it had been shown that a cDNA construct, *zic1ΔC*, containing a shorter truncation missing the C-terminal 36 amino acids (see Figure 2C), had enhanced activity compared to full-length cDNA, in transactivation, and *Xenopus* animal cap, and other in vivo assays.[27,38,39]

To search for further evidence that *ZIC1* mutations cause craniosynostosis, we screened a panel comprising 307 unrelated subjects with synostosis affecting any combination of sutures (including 45 and 112 with exclusively bilateral and unilateral coronal synostosis, respectively) and for whom no genetic diagnosis had been made. Initially we identified a single heterozygous nonsense mutation in this panel (subject 2: c.1204G>T encoding p.Glu402*); neither parent had the mutation, indicating that it had arisen de novo (sample relationships were confirmed by microsatellite analysis). We did not identify any *ZIC1* copy-number changes in this craniosynostosis panel by MLPA (data not shown). Later, we discovered by exome sequencing that a second individual included on the panel (subject 3) had the identical mutation, but present in mosaic state (see legend to Figure 2A for details). Strikingly, review of the phenotypes of subjects 2 and 3 revealed that both had bicoronal synostosis with severe brachycephaly (Figures 1B–1D); in addition, both had learning disability, which was milder in subject 3 who had the mosaic mutation. Review of CT brain scans showed that subject 2 had agenesis of corpus callosum and dilated lateral ventricles, but neither subject 2 or 3 had DWM (not shown).

In an attempt to further replicate these findings, we examined DNA samples collected at a second craniofacial unit (Rotterdam), specifically where the combination of both coronal synostosis and significant learning disability was present. Only three samples were available for analysis, which reflects the rarity of this combination of phenotypes; remarkably, however, dideoxy sequencing showed that one of these samples harbored a heterozygous nonsense mutation in *ZIC1* (c.1165C>T encoding p.Gln389*) at the codon adjacent to that affected in subject 1 (Figure 2A). This child had presented with bicoronal and unilateral lambdoid synostosis (Figures 1E and 1F) and had significant learning problems (Table 1). An MRI scan identified several cerebral anomalies including a short corpus callosum, mildly enlarged lateral ventricles, peaked tentorium, hypoplastic pons, and cerebellum with prominent cerebellar folia and enlarged foramen magnum with signal void near the cervical cord (Figure 1G).

The final family (family 5) consisted of six affected individuals in three generations (Figure 1H). Two cousins (subjects 5:III.3 and 5:III.6) had bicoronal synostosis (Figures 1I and 1J) and a further individual (subject 5:III.1, a half-brother of 5:III.3) had a DWM but no craniosynostosis (not shown). All three, and their respective mothers (5:II.2 and 5:II.4), had mild learning disability. Whole genome sequencing of four individuals (subjects 5:II.2, 5:II.3, 5:III.3, 5:III.6) identified a heterozygous variant in *ZIC1*, c.1198G>C encoding p.Gly400Arg, present in the three affected individuals (5:II.2, 5:III.3, 5:III.6) but not in the unaffected spouse (5:II.3). These results were confirmed by dideoxy sequencing, which showed that the variant was also present in 5:III.1 (the individual with DWM) and in 5:II.4 (the obligate transmitting mother) but not in her two unaffected children (5:III.4

Table 1. Phenotypic Features of Individuals with *ZIC1* Mutations

Subject ID	Reference ID	Gender	Mutation (cDNA) and Alteration (Protein)	Cranial Sutures	Number of Major Craniofacial Procedures	Other Brain Abnormalities on CT/MRI Scanning	Strabismus/Ptosis	Learning Disability	Other Major Clinical Features
1	4447	M	c.1163C>A (p.Ser388*)	bicoronal synostosis	3	abnormal configuration of ventricles and corpus callosum	–	moderate-severe	scoliosis, foreskin stricture
2	4098	F	c.1204G>T (p.Glu402*)	bicoronal synostosis	0	agenesis of corpus callosum, dilated lateral ventricles	divergent strabismus	moderate	scoliosis
3	4133/5847	M	c.1204G>T (p.Glu402*)[a]	bicoronal synostosis, bony defect of sagittal suture	2	normal on CT scan	–	mild	–
4	12D11570	M	c.1165C>T (p.Gln389*)	bicoronal synostosis, partial R lambdoid synostosis, bony defect of metopic and sagittal sutures	1	mildly enlarged lateral ventricles, shortened corpus callosum, hypoplastic pons, enlarged foramen magnum	strabismus sursoadductorius	moderate-severe	–
5:II.2	12D15615	F	c.1198G>C (p.Gly400Arg)	brachycephaly, delayed closure anterior fontanelle	0	atrophy of the rostral part of the cerebellum and pons	–	mild	–
5:II.4	08D1850	F	c.1198G>C (p.Gly400Arg)	plagiocephaly	0	reduced dorsum of pons, minor posterior fossa abnormalities	strabismus correction, ptosis L eye	below average	–
5:III.1	14D6457	M	c.1198G>C (p.Gly400Arg)	delayed closure anterior fontanelle (4 years)	0	DWM	–	mild	50 dB sensorineural hearing loss R; spina bifida occulta; LHRH deficiency
5:III.3	12D15613	M	c.1198G>C (p.Gly400Arg)	bicoronal synostosis, patent metopic, bilateral bony defect of lambdoid sutures	0	normal on CT scan	bilateral convergent strabismus + ptosis	mild	–
5:III.6	10D5797	M	c.1198G>C (p.Gly400Arg)	bicoronal synostosis, bilateral parietal foramina	1	reduced dorsum of pons, minor posterior fossa abnormalities	bilateral divergent strabismus + ptosis R eye	below average	–

[a]Mutation present in mosaic state.

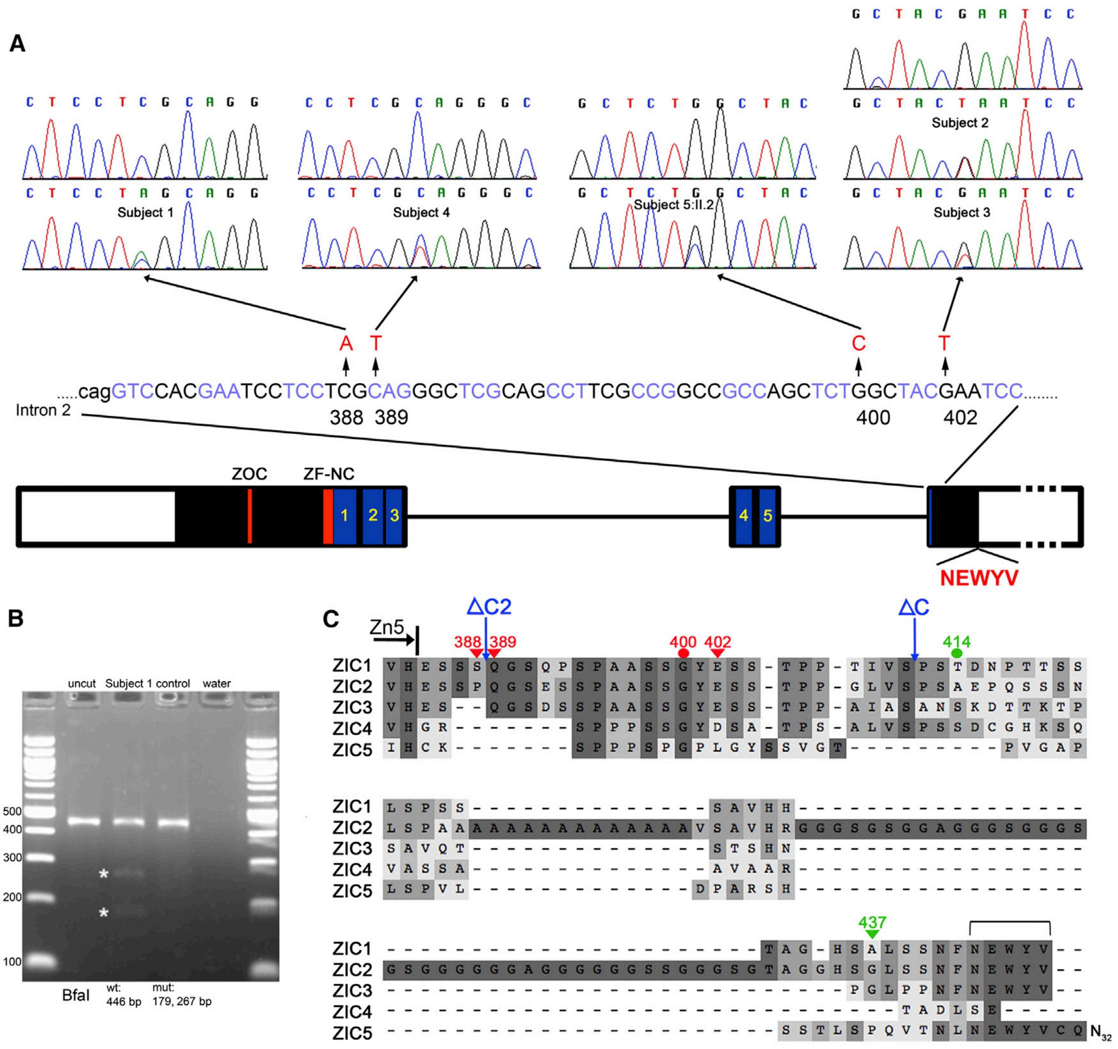

Figure 2. Molecular Genetic Analysis of Individuals with *ZIC1* Mutations

(A) Cartoon showing exon organization (white boxes denote non-coding regions) and previously identified conserved domains (Zic opa conserved motif [ZOC], zinc finger N-flanking conserved region [ZF-NC], and five zinc fingers [1–5, blue boxes]) of human *ZIC1*.[16] Also indicated is the C-terminal NEWYV motif conserved in all family members except *ZIC4*. Above the cartoon are the positions of the five independent *ZIC1* mutations described in this report, and dideoxy-sequence traces showing comparison of normal sequence (above) and mutant sequence (below). Note, in the case of subject 3, the mutation was not evident in the DNA sample (sourced from scalp fibroblasts; not shown) originally analyzed; however, in the exome sequence of DNA sourced from blood of the same individual, 63 of 183 (34%) reads showed the c.1204G>T mutation, which is also readily apparent on the dideoxy sequence. The relative heights of mutant and wild-type peaks differ between samples from subject 3 and subject 2, who is constitutionally heterozygous for the identical mutation, corroborating that in subject 3 the mutation is present in high-level mosaic state.

(B) Agarose gel analysis of *ZIC1* cDNA obtained from RNA extracted from scalp fibroblasts of subject 1 and digested with BfaI. The fragments yielded by digestion of the mutant allele are indicated with asterisks.

(C) Amino acid sequence encoded by 3′-terminal exon of *ZIC1* and comparison with the paralogous human proteins ZIC2-ZIC5, showing conservation including the NEWYV motif (bracket). The end of the fifth zinc finger (Zn5) is shown above the sequence, as are the positions of the four different pathogenic variants (red symbols) (triangle, nonsense; circle, missense) identified in this study. The positions at which the *Xenopus* constructs *zic1ΔC2* and *zic1ΔC* are truncated, relative to the human sequence, are indicated by blue arrows (note *zic1ΔC2* is equivalent to p.Gln389*). Additional human constructs tested in the *Xenopus* assay are indicated by green symbols.

and 5:III.5). This variant is absent from more than 120,000 alleles in the Exome Aggregation Consortium (ExAC). The combination of phenotypic features, the segregation of the variant, its location in the region where the previously identified nonsense mutations all clustered (Figure 2A), and absence in large databases of variation suggest that this variant is causative of the phenotype.

Functional Consequence of *ZIC1* Mutations in *Xenopus* Embryo Assay

The function of the *Xenopus zic1* ortholog has been studied in detail, where it was shown to act together with Pax3 as a key transcription factor required for the initiation of neural crest formation.[40] In *Xenopus* embryos, a signaling cascade has been proposed in which inhibition of bone

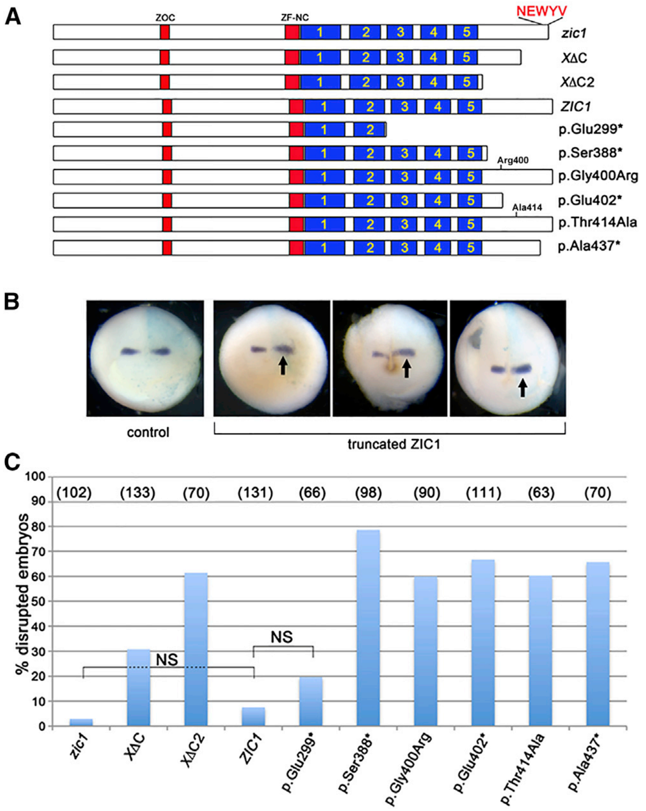

Figure 3. Analysis of Consequences of *ZIC1* Mutations in *Xenopus* Embryos

(A) Cartoon showing the structure and nomenclature of the cDNA constructs used in the experiment. The five zinc finger domains are highlighted in blue.

(B) *Xenopus en-2* expression after microinjection of *ZIC1* construct RNA (right side of each embryo). Arrows indicate widened, increased, or shifted *en-2* expression in three representative embryos.

(C) Quantification of effects of *ZIC1* mutations on *en-2* expression. The numbers of embryos used in each experiment are indicated in parentheses. Statistical comparisons between full-length *Xenopus zic1* and human *ZIC1* and between *ZIC1* and *ZIC1*-p.Glu299* were not significant (NS), whereas comparisons between all other mutants and corresponding full-length constructs were highly significant ($p < 10^{-8}$).

morphogenetic proteins (Bmps) activates *zic1*, which in turn activates members of the Wnt family including *wnt1*, which in turn activates the transcription factor *engrailed-2* (*en-2*).[27,38] A previously designed assay had shown that injection of *zic1ΔC* RNA encoding a truncated ZIC1 into a single cell of 2-cell stage embryos led to increased *en-2* expression in stage 15–17 *Xenopus* embryos, whereas injection of wild-type *zic1* construct had no effect.[27] We next examined the activity of human *ZIC1* and various mutant constructs with this assay.

In initial experiments, we replicated the previously reported results of the *Xenopus* assay. Although very few (3%) embryos were disrupted by injection of the wild-type *zic1* construct, this increased to 31% using the previously published *zic1ΔC*. Moreover, a construct deleting almost the entire region C-terminal to the zinc fingers (*zic1ΔC2*; Figure 2C) showed an even higher proportion (61%) of disrupted embryos (Figure 3). Using constructs encoding human ZIC1, injection of full-length *ZIC1* RNA yielded only 8% disrupted embryos, but this was increased to 79% and 68% with constructs corresponding to two of the observed truncations, p.Ser388* (subject 1) and p.Glu402* (subjects 2 and 3), respectively. Strikingly, a similar magnitude of effect (66%) was found with the most C-terminal truncation construct (p.Ala437*, which does not correspond to an observed mutation), highlighting the importance of the terminal 11 amino acids, which includes a 5-amino-acid motif (NEWYV) of unknown function that is conserved in human ZIC2, ZIC3 (isoform A), and ZIC5 (Figure 2C) but is not present in any other human protein. Two constructs encoding missense substitutions were studied with the same assay: p.Gly400Arg present in family 5 and p.Thr414Ala, corresponding to a rare SNP (dbSNP rs143292136), present in 104/121,380 alleles in ExAC, which we had identified in a child with bicoronal synostosis and her unaffected father (data not shown); 60% of embryos were disrupted in both cases. Although producing a slightly milder effect than the nonsense mutations, these results suggest that residues in the C-terminal domain in addition to the NEWYV motif contribute to function. Importantly, using a truncated control construct (p.Glu299*) missing the last three of the five zinc fingers, a much lower proportion of embryos (20%) was disrupted, which did not differ significantly from the full-length *ZIC1* RNA (Figure 3C). This indicates that intact zinc fingers are required to induce consistently abnormal *en-2* expression in this assay.

Zic1 Expression in Mouse Embryos

Of note in the above experiments, the effect of the ZIC1 C-terminal mutants was to increase the expression of the *Xenopus* target gene *en-2* (Figures 3B and 3C). Given previous evidence that the paralogous gene *En1* is critical for early biogenesis of the murine coronal suture,[11] we asked whether *Zic1* might also be expressed in relevant cells. Although the neural pattern of *Zic1* expression[20,41] and loss-of-function phenotypes[19,20] were previously described in the mouse, no evidence has linked *Zic1* expression to coronal suture development. Therefore, we analyzed expression patterns of *Zic1* in embryonic mouse heads between E11.5 and E17.5. Between E11.5 and E12.5, a distinct domain of *Zic1* expression was observed in the supraorbital region and cephalic mesoderm, which appeared to precede and partly overlap *En1* expression (Figure 4). By contrast, no *Zic1* expression was observed in the calvaria at E14.5 and E17.5 (not shown).

Discussion

Given the homology to one of the classical early patterning genes of the *Drosophila* embryo, the function of the vertebrate Zic family has been the focus of sustained

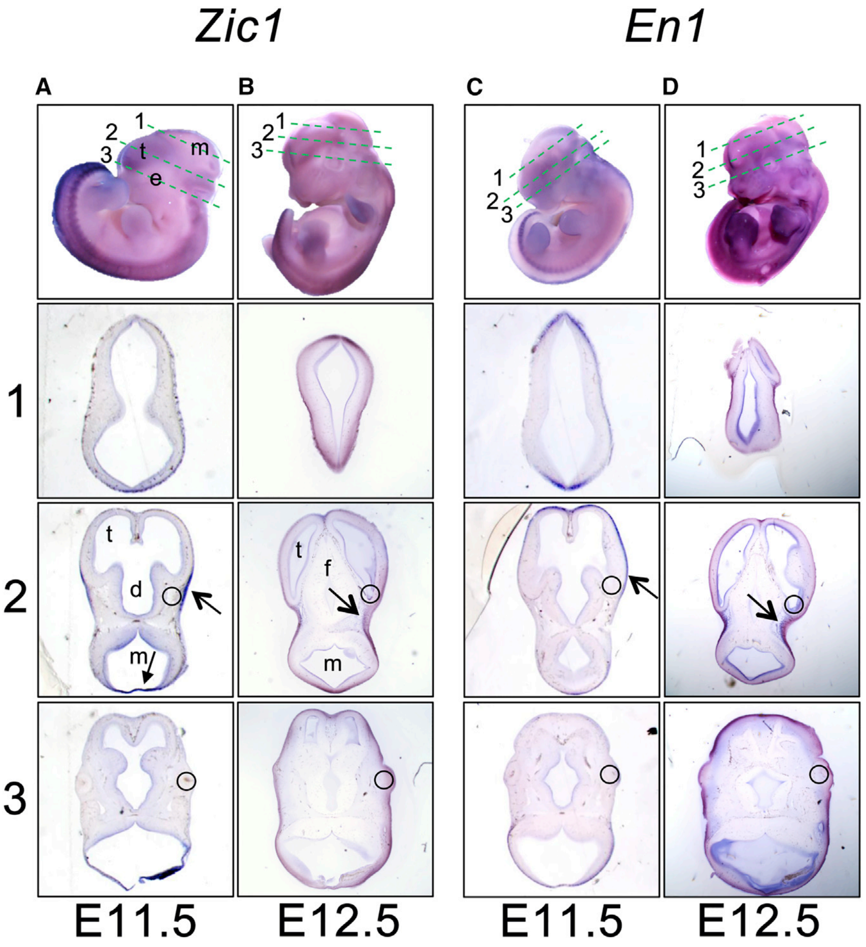

Figure 4. Expression of *Zic1* in E11.5–E12.5 Mouse Embryos Analyzed by RNA In Situ Hybridization and Comparison with *En1*

Panels show comparison of *Zic1* (A, B) and *En1* (C, D) expression at E11.5 (A, C) and E12.5 (B, D). In each case the top panel shows the whole embryo and plane of sections 1, 2, and 3, which are illustrated in the three respective lower panels (e, eye; t, telencephalon; d, diencephalon; m, mesencephalon; f, forebrain; circles indicate relative position of the eye). Note strong expression of *Zic1* in supraorbital region (open arrow) at E11.5; other areas of expression are midbrain-hindbrain boundary (closed arrow), neural tube, and limb mesenchyme (A). At E12.5 *Zic1* expression is seen mainly in the cephalic mesenchyme, just posterior to the eye (B). By comparison, at E11.5 the expression of *En1* expression is relatively weak in the supraorbital region (stronger expression is seen in the apical ectodermal ridge of the limb and in the somitic mesoderm). By E12.5 *En1* expression has increased in the cephalic mesoderm.

interest. Highlighting their importance for development, mutations in the related genes *ZIC2* (MIM: 603073) and *ZIC3* (MIM: 300265) were previously described in holoprosencephaly (MIM: 609637)[42] and X-linked visceral heterotaxy (MIM: 306955),[43] respectively. In the case of *ZIC1*, the observation that heterozygous deletions in tandem with *ZIC4* cause DWM[18,36] has supported work in *Xenopus*[27,38,40] and mouse,[20,41] indicating key roles for the Zic1 ortholog in neurogenesis (in the mouse, deficiency of *Zic1* and *Zic4* contribute additively to cerebellar hypoplasia).[20] Our data now highlight a previously unsuspected subsidiary role for ZIC1 in early patterning events in the coronal suture.

The nonsense mutations of *ZIC1* present in subjects 1 to 4 all arose de novo (post-zygotically in the case of subject 3, who has an attenuated phenotype) and are associated with consistent features comprising bicoronal synostosis, moderate to severe learning disability, and subtle abnormalities in brain anatomy including variable deficiency of the corpus callosum and abnormal conformation of the ventricles and posterior fossa. The progressive scoliosis in two of these individuals is likely to be causally related to the mutation, because vertebral and thoracic defects were also observed in mouse mutants homozygous for the null mutation in *Zic1*.[44] In family 5, a heterozygous missense variant p.Gly400Arg, present in the same C-terminal region of the ZIC1 protein, segregated through three generations in six affected individuals. The phenotype in this family was variable, with two individuals having documented bicoronal synostosis and another a DWM. Associated learning disabilities were less severe than in subjects with truncations. Interestingly, MRI scans showed that several individuals from this family not formally diagnosed with DWM nevertheless had more subtle abnormalities in the conformation of the posterior fossa (Figure 1J). Hence the cerebral features were reminiscent of, but milder than, the malformations described in the previous cases with contiguous *ZIC1*-*ZIC4* deletions. In contrast to many craniosynostosis syndromes, no diagnostic limb anomalies were apparent in any of the affected individuals.

In assessing the association of *ZIC1* mutations with craniosynostosis, two features are particularly striking: the highly localized distribution of the mutations and the severity of the phenotype. All five mutations predict protein alterations within a 15-residue stretch encoded by the final exon. The occurrence of distinct phenotypes and/or patterns of inheritance associated with truncations localized to the terminal exon is a well-recognized indication that escape from nonsense-mediated decay might be responsible.[37] Indeed, we were able to demonstrate that the transcript carrying the nonsense mutation was stable in a fibroblast cell line from the individual (subject 1) with the most N-terminal truncation (Figure 2B). The qualitative difference in phenotype associated with

heterozygous truncating mutations, compared to previously reported heterozygous deletions,[18,36] supports a gain-of-function mechanism (see also below). In the four case subjects with truncating mutations, the coronal synostosis was always bilateral and associated with marked brachycephaly (Figures 1A–1G), consistent with a severe, early effect on cranial suture formation; localized sutural ossification defects also occurred frequently. Craniosynostosis accompanied by DWM is rare, although an association with sagittal synostosis was reported previously.[45]

To investigate the mechanism by which these mutations could disturb coronal suture biogenesis, we first explored their functional effect in a previously established *Xenopus* assay. Injection of several constructs truncated at different positions in the C-terminal region led to disrupted *en-2* expression in a majority of treated embryos, provided that the zinc finger domain remained intact (Figure 3). Mostly the disrupted expression pattern involved combinations of upregulation, shifting, and expansion of *en-2* expression (for examples see Figure 3B), although with the larger truncations retaining all Zn fingers, some embryos showed reduced *en-2* expression. The *zic1ΔC* construct has been used previously in *Xenopus* to obtain enhanced biological responses to *zic1* in several assays in which sensitization of the ectoderm to Bmp inhibition led to activation of genes expressed in the neural crest and neural tube; this was interpreted as showing that the C-terminal region has negative regulatory activity.[38] The biological basis of this activity remains unknown; based on the enhanced *en-2* expression associated with both the very C-terminal truncation p.Ala437* and the two missense substitutions, this might involve protein interaction over several parts of the ZIC1 C-terminal region, which shows several patches that are conserved between multiple ZIC paralogs (Figure 2C). Although the *Xenopus* assay supports the genetic evidence that the c.1198G>C (p.Gly400Arg) substitution is causative of the phenotype in family 5, a cautionary note is provided by the finding that p.Thr414Ala also disrupted *en-2* expression, because this variant occurs too frequently (at 1 in 1,167 alleles) to be penetrant for craniosynostosis in more than a small proportion of individuals who carry this variant.

In a second approach to understand the pathogenic mechanisms, we asked whether the pattern of *Zic1* expression was consistent with a specific role in coronal suture biogenesis. We found a previously undescribed, transient zone of *Zic1* expression in the supraorbital region at E11.5 (Figure 4) that is spatially and temporally overlapping with that of *En1* (Figure 4) and is consistent with an early instructive role for *Zic1* in the supraorbital regulatory center; combining these observations with the finding of increased *en-2* expression driven by mutant ZIC1 constructs in the *Xenopus* experiments, we propose that the coronal synostosis phenotype associated with the human mutations might be attributable to alteration of *EN1* expression in the supraorbital regulatory center, thus disrupting the patterning of the coronal suture at a very early stage in its development. The induction of *engrailed* expression by *ZIC1* orthologs is well characterized in both *Drosophila*[15,46] and *Xenopus*[38] and is thought to act through the *Wnt* signaling pathway.[27] A further component of this signaling network is likely to be *Lmx1b*, which encodes a LIM-homeodomain protein and is upregulated in early neural crest of *Xenopus*.[47] In the mouse, *Lmx1b* is prominently expressed in the supraorbital region at E11.5, and homozygous mutants have severely abnormal cranial sutures.[48] In humans, heterozygous mutations in *LMX1B* usually cause nail-patella syndrome (MIM: 161200),[49] but a specific missense mutation in the N-terminal arm of the homeodomain has been associated with craniosynostosis.[1]

Putting the evidence together, we propose that the *ZIC1* mutations we have described serendipitously uncover an important role for this transcription factor in early lineage commitment at the supraorbital regulatory center[7] and that *En1* is likely to represent a key target gene. This will stimulate further work to define more precisely the role of *Zic1* in the coronal suture and to delineate the function of the conserved C-terminal domain. In *ZIC2*, constructs with holoprosencephaly-associated mutations C-terminal to the zinc fingers showed variable loss of transactivation activity,[50] whereas in *ZIC3*, the single missense mutation beyond the zinc fingers reported to date (in a simplex case subject with congenital heart disease) was uniquely associated with increased transactivation activity.[51] Collectively these data highlight the distinct properties of the C-terminal domain of ZIC proteins. The sequence conservation identified in this region (Figure 2C) suggests that a shared but currently unexplored mechanism exists for their regulatory function. Finally, although *ZIC1* mutations are rare because the genetic target is localized, the complications for intellectual and skeletal development are more serious than is usually the case with coronal synostosis; therefore, genetic testing is recommended when the more common diagnostic possibilities (involving mutations in *TWIST1*, *FGFR3*, *FGFR2*, and *TCF12*)[3,4] have been excluded.

Supplemental Data

Supplemental Data include two tables, supplemental case reports, and WGS500 member list and can be found with this article online at http://dx.doi.org/10.1016/j.ajhg.2015.07.007.

Acknowledgments

We thank Michael Parker and Julie Phipps for assistance with subject recruitment, Sue Butler for cell culture work, and Geoff Maher and Yan Zhou for preparing and running the PGM libraries. This work was funded by the National Science Foundation (DBI-1309250 to J.F. and IOS-0846168 to C.S.M.), the Wellcome Trust (Project Grant 093329 to A.O.M.W. and S.R.F.T., Senior Investigator award 102731 to A.O.M.W.), and the Oxford NIHR Biomedical Research Centre. Members of the 500 Whole-Genome Sequences (WGS500) Consortium are listed in the Supplemental Data.

Received: May 26, 2015
Accepted: July 14, 2015
Published: September 3, 2015

Web Resources

The URLs for data presented herein are as follows:

1000 Genomes, http://browser.1000genomes.org
dbSNP, http://www.ncbi.nlm.nih.gov/projects/SNP/
ExAC Browser, http://exac.broadinstitute.org/
NHLBI Exome Sequencing Project (ESP) Exome Variant Server, http://evs.gs.washington.edu/EVS/
OMIM, http://www.omim.org/
RefSeq, http://www.ncbi.nlm.nih.gov/RefSeq

References

1. Wilkie, A.O.M., Byren, J.C., Hurst, J.A., Jayamohan, J., Johnson, D., Knight, S.J.L., Lester, T., Richards, P.G., Twigg, S.R.F., and Wall, S.A. (2010). Prevalence and complications of single-gene and chromosomal disorders in craniosynostosis. Pediatrics *126*, e391–e400.
2. Lajeunie, E., Le Merrer, M., Bonaïti-Pellie, C., Marchac, D., and Renier, D. (1995). Genetic study of nonsyndromic coronal craniosynostosis. Am. J. Med. Genet. *55*, 500–504.
3. Johnson, D., and Wilkie, A.O.M. (2011). Craniosynostosis. Eur. J. Hum. Genet. *19*, 369–376.
4. Sharma, V.P., Fenwick, A.L., Brockop, M.S., McGowan, S.J., Goos, J.A., Hoogeboom, A.J., Brady, A.F., Jeelani, N.O., Lynch, S.A., Mulliken, J.B., et al.; 500 Whole-Genome Sequences (WGS500) Consortium (2013). Mutations in *TCF12*, encoding a basic helix-loop-helix partner of TWIST1, are a frequent cause of coronal craniosynostosis. Nat. Genet. *45*, 304–307.
5. Jiang, X., Iseki, S., Maxson, R.E., Sucov, H.M., and Morriss-Kay, G.M. (2002). Tissue origins and interactions in the mammalian skull vault. Dev. Biol. *241*, 106–116.
6. Yoshida, T., Vivatbutsiri, P., Morriss-Kay, G., Saga, Y., and Iseki, S. (2008). Cell lineage in mammalian craniofacial mesenchyme. Mech. Dev. *125*, 797–808.
7. Twigg, S.R.F., and Wilkie, A.O.M. (2015). A genetic-pathophysiological framework for craniosynostosis. Am. J. Hum. Genet. *97*, this issue, 359–377.
8. Merrill, A.E., Bochukova, E.G., Brugger, S.M., Ishii, M., Pilz, D.T., Wall, S.A., Lyons, K.M., Wilkie, A.O.M., and Maxson, R.E., Jr. (2006). Cell mixing at a neural crest-mesoderm boundary and deficient ephrin-Eph signaling in the pathogenesis of craniosynostosis. Hum. Mol. Genet. *15*, 1319–1328.
9. Hajihosseini, M.K., Duarte, R., Pegrum, J., Donjacour, A., Lana-Elola, E., Rice, D.P., Sharpe, J., and Dickson, C. (2009). Evidence that Fgf10 contributes to the skeletal and visceral defects of an Apert syndrome mouse model. Dev. Dyn. *238*, 376–385.
10. Holmes, G., Rothschild, G., Roy, U.B., Deng, C.X., Mansukhani, A., and Basilico, C. (2009). Early onset of craniosynostosis in an Apert mouse model reveals critical features of this pathology. Dev. Biol. *328*, 273–284.
11. Deckelbaum, R.A., Holmes, G., Zhao, Z., Tong, C., Basilico, C., and Loomis, C.A. (2012). Regulation of cranial morphogenesis and cell fate at the neural crest-mesoderm boundary by engrailed 1. Development *139*, 1346–1358.
12. Ting, M.C., Wu, N.L., Roybal, P.G., Sun, J., Liu, L., Yen, Y., and Maxson, R.E., Jr. (2009). *EphA4* as an effector of *Twist1* in the guidance of osteogenic precursor cells during calvarial bone growth and in craniosynostosis. Development *136*, 855–864.
13. Deckelbaum, R.A., Majithia, A., Booker, T., Henderson, J.E., and Loomis, C.A. (2006). The homeoprotein engrailed 1 has pleiotropic functions in calvarial intramembranous bone formation and remodeling. Development *133*, 63–74.
14. Ali, R.G., Bellchambers, H.M., and Arkell, R.M. (2012). Zinc fingers of the cerebellum (Zic): transcription factors and cofactors. Int. J. Biochem. Cell Biol. *44*, 2065–2068.
15. Benedyk, M.J., Mullen, J.R., and DiNardo, S. (1994). *odd-paired*: a zinc finger pair-rule protein required for the timely activation of *engrailed* and *wingless* in *Drosophila* embryos. Genes Dev. *8*, 105–117.
16. Aruga, J., Kamiya, A., Takahashi, H., Fujimi, T.J., Shimizu, Y., Ohkawa, K., Yazawa, S., Umesono, Y., Noguchi, H., Shimizu, T., et al. (2006). A wide-range phylogenetic analysis of Zic proteins: implications for correlations between protein structure conservation and body plan complexity. Genomics *87*, 783–792.
17. Merzdorf, C.S. (2007). Emerging roles for *zic* genes in early development. Dev. Dyn. *236*, 922–940.
18. Grinberg, I., Northrup, H., Ardinger, H., Prasad, C., Dobyns, W.B., and Millen, K.J. (2004). Heterozygous deletion of the linked genes *ZIC1* and *ZIC4* is involved in Dandy-Walker malformation. Nat. Genet. *36*, 1053–1055.
19. Aruga, J., Minowa, O., Yaginuma, H., Kuno, J., Nagai, T., Noda, T., and Mikoshiba, K. (1998). Mouse *Zic1* is involved in cerebellar development. J. Neurosci. *18*, 284–293.
20. Blank, M.C., Grinberg, I., Aryee, E., Laliberte, C., Chizhikov, V.V., Henkelman, R.M., and Millen, K.J. (2011). Multiple developmental programs are altered by loss of *Zic1* and *Zic4* to cause Dandy-Walker malformation cerebellar pathogenesis. Development *138*, 1207–1216.
21. Eley, K.A., Johnson, D., Wilkie, A.O.M., Jayamohan, J., Richards, P., and Wall, S.A. (2012). Raised intracranial pressure is frequent in untreated nonsyndromic unicoronal synostosis and does not correlate with severity of phenotypic features. Plast. Reconstr. Surg. *130*, 690e–697e.
22. Taylor, J.C., Martin, H.C., Lise, S., Broxholme, J., Cazier, J.-B., Rimmer, A., Kanapin, A., Lunter, G., Fiddy, S., Allan, C., et al. (2015). Factors influencing success of clinical genome sequencing across a broad spectrum of disorders. Nat. Genet. *47*, 717–726.
23. Rimmer, A., Phan, H., Mathieson, I., Iqbal, Z., Twigg, S.R.F., Wilkie, A.O.M., McVean, G., and Lunter, G.; WGS500 Consortium (2014). Integrating mapping-, assembly- and haplotype-based approaches for calling variants in clinical sequencing applications. Nat. Genet. *46*, 912–918.
24. Lunter, G., and Goodson, M. (2011). Stampy: a statistical algorithm for sensitive and fast mapping of Illumina sequence reads. Genome Res. *21*, 936–939.
25. Drmanac, R., Sparks, A.B., Callow, M.J., Halpern, A.L., Burns, N.L., Kermani, B.G., Carnevali, P., Nazarenko, I., Nilsen, G.B., Yeung, G., et al. (2010). Human genome sequencing using unchained base reads on self-assembling DNA nanoarrays. Science *327*, 78–81.
26. Carnevali, P., Baccash, J., Halpern, A.L., Nazarenko, I., Nilsen, G.B., Pant, K.P., Ebert, J.C., Brownley, A., Morenzoni, M., Karpinchyk, V., et al. (2012). Computational techniques for

human genome resequencing using mated gapped reads. J. Comput. Biol. *19*, 279–292.

27. Merzdorf, C.S., and Sive, H.L. (2006). The *zic1* gene is an activator of Wnt signaling. Int. J. Dev. Biol. *50*, 611–617.
28. Sive, H.L., Hattori, K., and Weintraub, H. (1989). Progressive determination during formation of the anteroposterior axis in *Xenopus laevis*. Cell *58*, 171–180.
29. Nieuwkoop, P.D., and Faber, J. (1967). Normal Table of *Xenopus laevis* (daudin) (Amsterdam: North-Holland Publishing Co).
30. Kolm, P.J., and Sive, H.L. (1995). Regulation of the *Xenopus* labial homeodomain genes, *HoxA1* and *HoxD1*: activation by retinoids and peptide growth factors. Dev. Biol. *167*, 34–49.
31. Harland, R.M. (1991). In situ hybridization: an improved whole-mount method for *Xenopus* embryos. Methods Cell Biol. *36*, 685–695.
32. Brivanlou, A.H., and Harland, R.M. (1989). Expression of an engrailed-related protein is induced in the anterior neural ectoderm of early *Xenopus* embryos. Development *106*, 611–617.
33. Wilkinson, D.G. (1998). *In situ* Hybridization: A Practical Approach (Oxford, New York: Oxford University Press).
34. Gaston-Massuet, C., Henderson, D.J., Greene, N.D., and Copp, A.J. (2005). *Zic4*, a zinc-finger transcription factor, is expressed in the developing mouse nervous system. Dev. Dyn. *233*, 1110–1115.
35. Wurst, W., Auerbach, A.B., and Joyner, A.L. (1994). Multiple developmental defects in *Engrailed-1* mutant mice: an early mid-hindbrain deletion and patterning defects in forelimbs and sternum. Development *120*, 2065–2075.
36. Ferraris, A., Bernardini, L., Sabolic Avramovska, V., Zanni, G., Loddo, S., Sukarova-Angelovska, E., Parisi, V., Capalbo, A., Tumini, S., Travaglini, L., et al.; Italian CBCD Study Group (2013). Dandy-Walker malformation and Wisconsin syndrome: novel cases add further insight into the genotype-phenotype correlations of 3q23q25 deletions. Orphanet J. Rare Dis. *8*, 75.
37. Khajavi, M., Inoue, K., and Lupski, J.R. (2006). Nonsense-mediated mRNA decay modulates clinical outcome of genetic disease. Eur. J. Hum. Genet. *14*, 1074–1081.
38. Kuo, J.S., Patel, M., Gamse, J., Merzdorf, C., Liu, X., Apekin, V., and Sive, H. (1998). Opl: a zinc finger protein that regulates neural determination and patterning in *Xenopus*. Development *125*, 2867–2882.
39. Li, S., Shin, Y., Cho, K.W., and Merzdorf, C.S. (2006). The *Xfeb* gene is directly upregulated by Zic1 during early neural development. Dev. Dyn. *235*, 2817–2827.
40. Milet, C., Maczkowiak, F., Roche, D.D., and Monsoro-Burq, A.H. (2013). Pax3 and Zic1 drive induction and differentiation of multipotent, migratory, and functional neural crest in *Xenopus* embryos. Proc. Natl. Acad. Sci. USA *110*, 5528–5533.
41. Nagai, T., Aruga, J., Takada, S., Günther, T., Spörle, R., Schughart, K., and Mikoshiba, K. (1997). The expression of the mouse *Zic1, Zic2*, and *Zic3* gene suggests an essential role for *Zic* genes in body pattern formation. Dev. Biol. *182*, 299–313.
42. Brown, S.A., Warburton, D., Brown, L.Y., Yu, C.Y., Roeder, E.R., Stengel-Rutkowski, S., Hennekam, R.C.M., and Muenke, M. (1998). Holoprosencephaly due to mutations in *ZIC2*, a homologue of *Drosophila odd-paired*. Nat. Genet. *20*, 180–183.
43. Gebbia, M., Ferrero, G.B., Pilia, G., Bassi, M.T., Aylsworth, A., Penman-Splitt, M., Bird, L.M., Bamforth, J.S., Burn, J., Schlessinger, D., et al. (1997). X-linked situs abnormalities result from mutations in *ZIC3*. Nat. Genet. *17*, 305–308.
44. Aruga, J., Mizugishi, K., Koseki, H., Imai, K., Balling, R., Noda, T., and Mikoshiba, K. (1999). *Zic1* regulates the patterning of vertebral arches in cooperation with *Gli3*. Mech. Dev. *89*, 141–150.
45. Braddock, S.R., Jones, K.L., Superneau, D.W., and Jones, M.C. (1993). Sagittal craniosynostosis, Dandy-Walker malformation, and hydrocephalus: a unique multiple malformation syndrome. Am. J. Med. Genet. *47*, 640–643, discussion 644.
46. DiNardo, S., and O'Farrell, P.H. (1987). Establishment and refinement of segmental pattern in the *Drosophila* embryo: spatial control of *engrailed* expression by pair-rule genes. Genes Dev. *1*, 1212–1225.
47. Plouhinec, J.L., Roche, D.D., Pegoraro, C., Figueiredo, A.L., Maczkowiak, F., Brunet, L.J., Milet, C., Vert, J.P., Pollet, N., Harland, R.M., and Monsoro-Burq, A.H. (2014). Pax3 and Zic1 trigger the early neural crest gene regulatory network by the direct activation of multiple key neural crest specifiers. Dev. Biol. *386*, 461–472.
48. Chen, H., Ovchinnikov, D., Pressman, C.L., Aulehla, A., Lun, Y., and Johnson, R.L. (1998). Multiple calvarial defects in *lmx1b* mutant mice. Dev. Genet. *22*, 314–320.
49. Dreyer, S.D., Zhou, G., Baldini, A., Winterpacht, A., Zabel, B., Cole, W., Johnson, R.L., and Lee, B. (1998). Mutations in *LMX1B* cause abnormal skeletal patterning and renal dysplasia in nail patella syndrome. Nat. Genet. *19*, 47–50.
50. Brown, L., Paraso, M., Arkell, R., and Brown, S. (2005). *In vitro* analysis of partial loss-of-function ZIC2 mutations in holoprosencephaly: alanine tract expansion modulates DNA binding and transactivation. Hum. Mol. Genet. *14*, 411–420.
51. Cowan, J., Tariq, M., and Ware, S.M. (2014). Genetic and functional analyses of *ZIC3* variants in congenital heart disease. Hum. Mutat. *35*, 66–75.

The American Journal of Human Genetics
Supplemental Data

Gain-of-Function Mutations in *ZIC1* Are Associated with Coronal Craniosynostosis and Learning Disability

Stephen R.F. Twigg, Jennifer Forecki, Jacqueline A.C. Goos, Ivy C.A. Richardson, A. Jeannette M. Hoogeboom, Ans M.W. Van den Ouweland, Sigrid M.A. Swagemakers, Maarten H. Lequin, Daniel Van Antwerp, Simon J. McGowan, Isabelle Westbury, Kerry A. Miller, Steven A. Wall, WGS500 Consortium, Peter J. van der Spek, Irene M.J. Mathijssen, Erwin Pauws, Christa S. Merzdorf, and Andrew O.M. Wilkie

Dominant/de novo variants							
	chr	position	from	to	gene	type	comment
1	3	147131157	C	A	*ZIC1*	Stop	*de novo*
2	9	140777194	A	AGCGGCT	*CACNA1B)*	splicing	maternal
3	13	21746477	C	CGTGTA	*SKA3*	splicing	artefact/present in multiple samples
4	1	39879157	A	C	*KIAA0754*	nonsynonymous	artefact/present in multiple samples
5	1	145343385	C	A	*NBPF10*	nonsynonymous	present in multiple samples
6	1	144823868	T	G	*NBPF9*	nonsynonymous	present in multiple samples
7	1	144815968	G	A	*NBPF9*	nonsynonymous	present in multiple samples
8	1	7890026	A	G	*PER3*	nonsynonymous	artefact/present in multiple samples
9	4	88537261	A	T	*DSPP*	nonsynonymous	maternal
10	4	85998	T	A	*ZNF595*	nonsynonymous	present in multiple samples
11	6	168377071	G	A	*HGC6.3*	nonsynonymous	paternal
12	6	168377029	G	A	*HGC6.3*	nonsynonymous	paternal
13	8	144940209	T	C	*EPPK1*	nonsynonymous	artefact
14	12	50745703	T	G	*FAM186A*	nonsynonymous	artefact
15	15	82637061	C	T	*GOLGA6L10*	nonsynonymous	artefact/present in multiple samples
16	15	20740611	C	T	*GOLGA6L6*	nonsynonymous	paternal
17	16	22545467	C	T	*LOC100132247*	nonsynonymous	artefact/present in multiple samples
18	16	88599705	G	C	*ZFPM1*	nonsynonymous	artefact/present in multiple samples
19	16	88599703	T	C	*ZFPM1*	nonsynonymous	artefact/present in multiple samples
20	17	18544392	T	C	*TBC1D28*	nonsynonymous	maternal
21	17	15620506	G	A	*ZNF286A*	nonsynonymous	maternal
22	19	53553339	C	G	*ERVV-2*	nonsynonymous	paternal
23	19	56104423	A	C	*FIZ1*	nonsynonymous	paternal
24	X	8434191	G	A	*VCX3B*	nonsynonymous	present in multiple samples
25	1	144615246	A	AAG	*NBPF9*	frameshift insertion	artefact/present in multiple samples
26	19	14070706	A	AGGTGGG CCCAGGG CGGGCAG	*DCAF15*	frameshift insertion/splicing	artefact/present in multiple samples
27	Y	21154527	CTGCGTGGG	C	*CD24*	frameshift deletion	artefact/present in multiple samples
28	1	248524966	A	AGCTCTA CTTAGT	*OR2T4*	nonframeshift insertion	artefact/present in multiple samples
29	4	88535832	A	ATAGCAG TGACAGC AGCAG	*DSPP*	nonframeshift insertion	artefact/present in multiple samples
30	6	34857302	G	GGGCGGC	*ANKS1A*	nonframeshift insertion	paternal
31	11	8414087	G	GCCAGAA	*STK33*	nonframeshift insertion	present in both parents

32	12	125478381	C	CCTG	*BRI3BP*	nonframeshift insertion	present in multiple samples
33	17	72889649	C	CGTAGGT TCCATGG GCTCCGT A	*FADS6*	nonframeshift insertion	artefact/present in multiple samples
34	1	26608853	GCCGGGACCGGG ACCGGGACTGGG GCCGGGA	G	*UBXN11*	nonframeshift deletion	paternal and other samples
35	5	60628153	GGGCGGC	G	*ZSWIM6*	nonframeshift deletion	paternal
36	6	45390486	AGGCGGCGGCGG CGGCTGC	A	*RUNX2*	nonframeshift deletion	paternal and other samples
37	11	117789312	CGGGCTGGAGATG CCT	C	*TMPRSS13*	nonframeshift deletion	paternal and other samples
38	16	29821419	TGGCGGC	T	*MAZ*	nonframeshift deletion	paternal
39	17	43319434	TCCG	T	*FMNL1*	nonframeshift deletion	present in multiple samples
**Recessive variants**							
1	2	99013251	G	A	*CNGA3*	nonsynonymous	Homozygous; p.(Val540Ile)
2	2	152376170	A	G	*NEB*	splicing	paternal
	2	152530992	A	C	*NEB*	nonsynonymous/splicing	maternal

Table S1. Whole genome sequence analysis

Screening

	Primer sequence 5′→3′ (M13 tags in lowercase)		Pro duct (bp)	Amplification conditions[a]
Amplicon	Forward	Reverse		
Ex1a F/R	gtaaaacgacggccagt GCCGGGGCTCGCCCCGAGCAGCCACG	agcggataacaatttcacacagga ATCTGCCCGTTGACCACGTTAG	555	65°C + DMSO
Ex1b F/R	gtaaaacgacggccagt TTCCCCGGGCTTCACGAGCAGGCTGC	agcggataacaatttcacacagga GATCCCACCGAGGCTGCGTTTGTGCGACC	698	65°C + DMSO
Ex2 F/R	gtaaaacgacggccagt TTTTAAGCTTGCAAAGTGCTAATCCTG	agcggataacaatttcacacagga CCAAGAGAGCTCCTGCCTCAAAG	385	65°C
Ex3 F/R	gtaaaacgacggccagt GGGCTCCAAGGGGTCCAGGAGGAAGGG	agcggataacaatttcacacagga GTGTATACGTGTGTGATCAGTCTCTTAAATAGGG	404	65°C

Primers and amplification conditions for Subject 1 cDNA[b]

cDNA	Primer sequence 5′→3′		Product size (bp)	Amplification conditions[a]	Digest
Fragment	Forward	Reverse			
Exon 2-3	GGGAGAAGCCCTTCAAGTGCGAGTTTGAGGG	CGCAGGGTTCTTTCAGTAATGTTGTGTATAC	446	+ DMSO	BfaI(+)

Multiplex ligation-dependent probe amplification (MLPA)[c]

	Primer sequence 5′→3′		Product size (bp)
Probe	Forward	Reverse	
Exon 1	gggttccctaagggttggaCATCTGCTTCTGGGAGGAGTGTCCGCGCGAGGGCAAGCCCTTCAA AGCCAAA	TACAAACTGGTTAACCACATCCGCGTGCACACGtctagattggatcttgctggcac	127
Exon 2	gggttccctaagggttggaCAGTGCGAGTTTGAGGGCTGTGACCGGCGCTTCGCTA	ACAGCAGCGACCGCAAGAAGCACATGCCtctagattggatcttgctggcac	107
Exon 3	gggttccctaagggttggaGCCAACTGTTTGTACTGAATGGCAAGAATGTTCTAGTAAATGTGT ACCAA	AATGTGAATTACTTTGTACGATTACAGTCTCCACGTCGACCTAACCCtctagattggatcttgctggcac	139

cDNA cloning and mutagenesis

	Primer sequence 5′→3′		
	Forward	Reverse	
cDNA PCR[d]	CGC*CTCGAG*CAGCCACGATGCTCCTGG	CGATGTTTTGT*TCTAGA*TTTTAAACGTACCATTCG	1380 bp
S388*	GAATCCTCCT**A**GCAGGGCTCG	GTGGACCTTCATGTGTTTGC	
E402*	CTCTGGCTAC**T**AATCCTCCACGCCTCCC	CTGGCGGCCGGCGAAGGC	
S436*	CGGCCACAGTT**TA**GCTCTCTTCCAATTTTAAC	GCTGTGTGGTGGACTGCG	
E299*	GCACACAGGG**T**AGAAGCCCTT	GTCCTTTTGTGGATCTTTAAATTCTC	
T414A	ATCGTGTCTCCCTCC**G**CAGACAACCCGACCACA	TGTGGTCGGGTTGTCTG**C**GGAGGGAGACACGAT	
G400R	CCGGCCGCCAGCTCTC**G**CTACGAATCCTCCACG	CGTGGAGGATTCGTAG**C**GAGAGCTGGCGGCCGG	

cDNA Ion Torrent PGM sequencing[e]

	Primer sequence 5′→3′		
	Forward	Reverse	
ZIC1 PGM f/r	CGCTCTTCCGATCTCTGcacgagcgacaagccctatctttgcaag	TGCTCTTCCGATCTGACtaaggagcttgtggtcgggttgtctgtg	200 bp
A-BC1Rdfw	CCATCTCATCCCTGCGTGTCTCCGACTCAGacgagtgcgtCGCTCTTCCG ATCTCTG	CCTCTCTATGGGCAGTCGGTGATTGCTCTTCCGATCTGAC	P1-Rdrev

Table S2. Primers and amplification conditions. [a]DNA was obtained from whole blood samples by phenol-chloroform extraction and was amplified in a total volume of 20 µl containing 15 mM TrisHCl (pH 8.0), 50 mM KCl, 2.5 mM $MgCl_2$, 100 µM each dNTP, 0.4 µM primers, and 0.5 units of FastStart Taq (Roche) with or without 10% DMSO. Cycling conditions consisted of an 8 min denaturation step at 94°C, followed by 35 cycles of 94°C for 30 s, 65°C for 30 s and 72°C for 30s, with a final extension at 72°C for 10 min. [b]Mutation confirmation was carried out by PCR using the above conditions and indicated primers, followed by restriction digest of 8 µl of PCR product. [c]Multiplex-ligation-dependent probe amplification was performed using synthetic oligonucleotide probes designed to *ZIC1* according to protocols available from MRC-Holland: http://www.mrc-holland.com/pages/indexpag.html. Fragments were analyzed by capillary electrophoresis using an ABI 3130 containing POP-7 polymer. Peaks were visualized using Gene Mapper v3.7 (Applied Biosystems). Common PCR primer annealing sequences are shown in lower case, hybridizing sequences are shown in upper case and the 3' probe sequence is 5' phosphorylated.[d]cDNA amplification was performed using pCR4-Topo-ZIC1 (ThermoFisher) as template and PCR conditions as described above except that the high fidelity FastStart Taq (Roche) was used. Bold letters within the primer sequences are the mutated bases. The amplification product was cloned into pcDNA3 using XhoI and XbaI (sites are in italics in the primer sequences). [e]Amplification to generate templates for PGM sequencing was performed on fibroblast cDNA (see Methods) using primers PGM F and PGM R (*ZIC1* specific sequences are shown in lowercase) and the conditions as above except that the cycle number was 30. The product was diluted 1 in 100 and a second amplification carried out to add the Ion Torrent PGM adapters P1 and A with conditions as above except that high fidelity FastStart Taq (Roche) was used and cycle number was reduced to 8. Lowercase bases in the A-BC1-Rdfw primer correspond to barcode sequence.

Case reports

Subject 1

This male was the first child born to a healthy unrelated couple, aged 38.9 years (father) and 28.5 years (mother) at the time of birth. The mother had four healthy children by a previous relationship. A brother born subsequently had metopic synostosis but this is presumed to be coincidental since he had normal development and was negative for the *ZIC1* mutation.

During the pregnancy, renal dilatation had been noted at 22 weeks' gestation; a fetal blood sample showed a normal karyotype. The proband was born at 37 weeks' gestation by Caesarean section owing to persistent transverse lie, weighed 2.98 kg and was noted to have an abnormal head shape at birth. On assessment aged 20 weeks, his occipito-frontal circumference (OFC) was 38.5 (-3.5 SD). He was noted to have severe brachycephaly, a high forehead, downslanting palpebral fissures and an open posterior fontanelle. The remainder of the examination was normal except for a transverse palmar crease on the right hand. The computed tomography (CT) head scan showed bilateral coronal synostosis and no identifiable cerebral abnormality. He underwent a fronto-orbital advancement and remodelling procedure at the age of 7 months. By the age of 8 months he was noted to have developmental delay, with age-equivalent development at 4-5 month level. On formal assessment at 29 months using Bayley Scales of Infant Development (2nd Ed), his motor development was equivalent to 17 months and mental development to 12 months. Hearing and vision assessments were normal, although he previously required insertion of grommets. At 2.5 years his intracranial pressure (ICP) was measured because of concerns about slow head growth and delayed development, this was markedly increased (baseline 25-30 mm Hg, peaks up to 50 mm Hg) and he underwent a posterior release and remodelling procedure. By 3 years he was showing hyperactive behaviour, with sleep disturbance and head-banging. A repeat ICP measurement aged 4.6 years was again elevated with episodes of abnormal pressure in the 25-35 mm Hg range, and he underwent a posterior release and advancement procedure.

At the age of 5 years he was noted to have a stricture of his foreskin and was developing a scoliosis. By 8 years, the left sided thoraco-lumbar curve measured 50°, and he had extensive spina bifida occulta in his lower lumbar spine; he subsequently had spinal surgery at the age of 10 years. He started to develop intermittent outbursts of aggressive behaviour, but several measurements of ICP were normal. A magnetic resonance imaging (MRI) brain scan showed abnormal configuration of the ventricles and corpus callosum. His developmental progress was slow and he attended a school for children with special educational needs. Genetic testing for causes of learning disability including repeat karyotype, telomere screen, *FRAX* and *PW71B* methylation (for Prader-Willi/Angelman syndrome) were normal. Testing for craniosynostosis mutations in the research laboratory (*FGFR2*, *FGFR3*, *TWIST1*) was normal. On assessment at the age of 13 years, he continued to have violent outbursts and poor sleeping routine. He had no language and only limited communication using signs, but his gross motor skills and comprehension were less severely delayed. He showed autistic features, being dependent on routines and focused on individual tasks.

Subject 2

This female was the only child of healthy unrelated parents aged 34.4 years (father) and 31.7 years (mother). She was born at term by forceps delivery following a normal pregnancy and weighed 3.23 kg. Although well at birth she fed poorly and was referred at the age of 4 months for assessment of an unusual head shape and dysmorphic appearance with "almond-shaped eyes" and down-slanted palpebral fissures, when a skull radiograph showed craniosynostosis. A clinical geneticist noted a high forehead, large open anterior fontanelle, flat occiput, maxillary hypoplasia and normal extremities, and suggested a diagnosis of Crouzon syndrome. Her development was moderately delayed; she walked unsupported at 2 years and had a vocabulary of 30 words at 2.8 years. She attended special schools for children with moderate to severe learning disability. She had surgery for divergent strabismus aged 3 years. CT head scan at 9 years showed dilated lateral ventricles and agenesis of the corpus callosum. At the age of 13 years she was noted to have a kyphoscoliosis. Plain radiographs showed multiple abnormalities of the thoracic spine and ribs and spondylolisthesis at L5/S1. MRI scan aged 17 years confirmed the previous findings and demonstrated a retroverted odontoid peg impinging on the cranio-cervical junction. There was no deterioration on orthopaedic follow-up to the age of 21 years.

She was referred for craniofacial assessment at the age of 23 years. Her head circumference was 48.5 cm (-5 SD) and height 149 cm (-2.2 SD). She was noted to be markedly brachycephalic with a high, flat brow and low frontal hairline. The remainder of the examination was normal and did not reveal diagnostic features of any craniosynostosis syndrome. Genetic testing (*FGFR1*, *FGFR2*, *FGFR3*, *TWIST1*) was negative and the karyotype was normal. CT scanning was consistent with bicoronal synostosis and demonstrated dilatation of the trigone and temporal regions of the ventricles. No surgical intervention was undertaken in view of her stable state. Currently aged 36 years, she lives in supervised residential accommodation, is able to communicate in short sentences and wash, dress and feed herself, but requires help with cooking.

Subject 3

This boy was the first child born to healthy unrelated parents after a normal pregnancy and weighed 3.34 kg (-0.1 SD). He was noted to have brachycephaly shortly after birth and was referred for craniofacial assessment aged 6 weeks. At 19 weeks his OFC was 39.0 cm (-2.8 SD) and cephalic index was 1.24. He had a low frontal hairline and normal extremities; on clinical genetic assessment his appearance was considered consistent with either Muenke or Saethre-Chotzen syndrome, but genetic testing of *FGFR3* and *TWIST1* was normal. The CT scan at the age of 5 months showed bicoronal synostosis, a patent metopic suture, a large wormian bone in the position of the anterior fontanelle, and an ossification defect in the sagittal suture. He developed progressive turricephaly and underwent a posterior distraction procedure aged 8 months followed by fronto-orbital advancement and remodelling aged 2.4 years.

He had mild developmental delay with speech dysfluency, and occasional tantrums with changes in routine. Tests of vision and hearing were normal. At the age of 8 years he was attending a normal school but was receiving 1:1 educational support because of delayed learning. Array comparative genomic hybridisation was normal except for a 140 kb deletion

on chromosome 17 that was inherited from the mother and therefore presumed to be coincidental to the phenotype.

Subject 4

This boy was the fourth child of healthy unrelated parents aged 41 years (father) and 36 years (mother); an older sibling had trisomy 18. The proband was born at 38 weeks' gestation, weighing 4.27 kg. An altered skull shape was immediately apparent; on assessment by the plastic surgery department at the age of 2.5 months, this was clinically consistent with bilateral coronal synostosis and microcephaly (OFC 36 cm; -2.1 SD), but without significant exorbitism, ptosis or malformations of the extremities. 3D CT scan showed bilateral coronal synostosis and a partial right lambdoid synostosis; the metopic and mid-part of the sagittal suture were widely patent. A clinical diagnosis of Muenke or Saethre-Chotzen syndrome was considered, but appropriate genetic testing was negative. Fronto-orbital and supraorbital rim remodeling, and osteotomy of the right lambdoid suture were performed at the age of 7 months.

On reassessment at the age of one year, delayed development was apparent. An MRI brain scan at 16 months showed a short corpus callosum, mildly enlarged lateral ventricles, a peaked tentorium cerebelli, enlarged foramen magnum with hypoplastic pons and cerebellum with prominent cerebellar folia and signal void near the cervical cord. The left transverse and sigmoid sinuses, together with the jugular foramen were enlarged, whereas on the right the transverse and sigmoid sinuses were underdeveloped. Ophthalmological assessment at the age of 18 months showed esotropia of the right eye, latent nystagmus, strabismus sursoadductorius with V motility and bilateral granular pigmented retinae. Metabolic testing was normal. Adenotonsillectomy was performed at the age of 2 years because of recurrent tonsillitis and otitis media.

Development continued to be delayed and was formally assessed at the age of 34 months. Motor development was at 15 months equivalent, but no formal testing of mental development or speech was possible. His back was hyperpigmented and pedes plano valgi were present. At 4 years, speech and gait were noted to be ataxic and autistic spectrum disorder and attention deficit hyperactivity disorder were diagnosed. Optic nerve hypoplasia was noted, worse in the right eye in which vision was reduced to 30% of normal; the left eye was myopic (-2.25 diopters).

Subject 5:III.3 (proband)

This male was the second child born at 38 weeks' gestation to unrelated parents. His grandfather (I.1), mother (II.2) and half-brother (III.1) had a similar head shape (see below). He weighed 2.94 kg and was well except for neonatal jaundice. At 30 months a brachycephalic head shape was noted with OFC of 46 cm (-2.5 SD), associated with patent posterior fontanelle (3.3 cm), an asymmetrical face, beaked nose, a high palate, and downslant and ptosis of the eyes. No hearing deficits or malformations of the upper or lower extremities were present. A clinical diagnosis of Saethre-Chotzen syndrome was suggested.

His development was generally delayed; he started walking at the age of 2.5 years and had delayed speech. He was generally hypotonic with joint laxity. An electroencephalogram

(EEG) showed an irregular and diffuse disordered background pattern with accentuated frontal dysfunction. He had a bilateral convergent strabismus.

He was referred for plastic surgical assessment at the age of 3.5 years. The craniofacial features were as noted previously. Vision was normal with glasses. Skull radiographs showed bicoronal synostosis with increased thumbprinting of the frontal bones. CT head scanning demonstrated in addition a patent metopic suture, bilateral bony defect of the lambdoid suture, and normal ventricles and posterior fossa. No surgery was performed because of his age.

His most recent review was at the age of 19 years. He still complained of headaches. He had a mildly delayed development and lived under social supervision. His communication was normal. Mild flattening of the left supra-orbital region, mild asymmetry of the skull, and downslant and exorbitism of the eyes were present.

Subject 5:III.1

The older half-brother of the proband was born at 40 weeks' gestation with a weight of 4.00 kg and a length of 52 cm. He was well during infancy but was clumsy with mildly delayed developmental milestones (walked at 3 years, rode a bicycle at 6 years, swimming certificate at 12 years). He had a large anterior fontanelle that did not close until 4 years of age. Attention deficit disorder was diagnosed.

At the age of 8 years he was investigated for suspected hearing loss. He had persisting central hypotonia, was dysarthric, mildly dysphasic and dyspraxic, and was unable to heel-toe walk. Hyperpigmentation was noted over his back. He was found to have a 50 dB left-sided sensorineural hearing loss; auditory evoked potentials suggested a sensorineural cause. CT head scan showed cystic dilatation of the 4th ventricle communicating with the cisterna magna, and hypoplasia of the cerebellar vermis. The third and lateral ventricles and the foramina of Luschka and Magendi were normal. These features were consistent with a variant of Dandy-Walker malformation. Visual evoked potentials were normal and a radio-iodinated serum albumin brain scan suggested obstruction of drainage of the left lateral ventricle. Radiology of the spine showed hypolordosis of the cervical spine and spina bifida occulta with absent arches of the L4, L5 and S1 vertebrae.

During the latter part of childhood his symptoms persisted. At the age of 15 years LHRH deficiency was diagnosed because of pubertal delay and he was started on pulsatile LHRH therapy. On further assessment aged 16 years, his OFC was 53 cm (-1.96 SD) and hyperpigmentation of his back was present. An MRI scan confirmed the earlier CT findings and showed atrophy of the cerebellum, the brachium pontis and temporo-basal lobes with widened sulci.

Subject 5:II.2

Little detailed information is available on the mother of III-1 and III-3. She reported that as a child she had an enlarged late-closing fontanel, and had learning disability requiring her to repeat classes in both primary and secondary school. She suffered from vertigo and had a clumsy tandem gait. MRI scan showed atrophy of the rostral part of the cerebellum and pons, similar to, but less severe than, III-1.

The family was investigated for a suspected autosomal dominant cerebellar atrophy, but relevant genetic testing (*SCA1*, *SCA2*, *SCA3* and *SCA6*) was negative in both 5:II.2 and 5:III.1.

Subject 5:III.6

The proband's male cousin, the 3rd child in the sibship (the two older siblings were healthy) was born at 37 weeks' gestation after an uneventful pregnancy, weighing 3.35 kg. He was referred for a plastic surgery opinion at one month of age because of concerns about his head size. He was microcephalic (OFC 38.5cm at 3.5 months; -1.9 SD) and noted to have brachycephaly, hypoplastic supraorbital ridges, hypertelorism, mild ptosis of the right eye, downslanting palpebral fissures, divergent strabismus, a high narrow palate, normal ears and symmetrical defects palpable over the occipito-parietal region. Additional features noted were axial hypotonia (but with increased muscle tone of the upper extremities), mild tapering of the fingers, clinodactyly of the 4th and 5th toes and a sacral dimple. A clinical diagnosis of Saethre-Chotzen syndrome was suggested. 3D CT scanning at the age of 2 months showed bilateral coronal synostosis with bilateral large parietal foramina. Ultrasound of the brain was normal. A fronto-orbital advancement was performed at the age of 11 months. The divergent strabismus persisted and posterior tenotomies were performed at the age of 3 years and 5 months.

At the age of 13 years an MRI brain scan was performed. The fourth ventricle and both superior cerebellar peduncles were mildly enlarged, with a prominent great cerebral vein and inferior sagittal sinus. The posterior fossa was small with a peaked tentorium cerebelli, short and steep straight sinus with mild hypoplasia of the dorsal part of the pons and inferior part of the cerebellar vermis. There was no Chiari or Dandy-Walker malformation.

His schooling history is not documented. Formal psychological testing was performed at the age of 16 years. The WISCIII scores were full scale IQ 89, verbal IQ 96, performance IQ 84. The Child Behavior Checklist 6-18, Social Communication Questionnaire, and Children's Communication Checklist-2 NL were normal. The OFC was 55cm (0 SD). He had small teeth and wore orthodontic braces.

Subject 5:II.4

This individual is the sister of II.2 and mother of III.6. As a child she had a strabismus correction, but no other surgery. She had mildly delayed development with attention deficit, aberrant fine motor skills and clumsiness, but attended a normal school. Her hearing and balance were normal. Currently aged 53 years, she complains of persistent severe headaches, forgetfulness and dental problems. On examination her height was 1.62 m (-0.3 SD), she had brachymicrocephaly (OFC 51cm, -2.9 SD), vertical orbital dystopia, ptosis of the left upper eyelid, midface hypoplasia, nasal deviation, and facial and occlusal asymmetry with narrow maxilla, crowding of the upper front teeth, absence of the lower right first molar and upper right canine, and prominent crura of both ears. The hands and feet were normal. MRI brain scan showed asymmetry of the skull base and head shape, an enlarged foramen magnum, a peaked tentorium cerebelli, mildly hypoplastic inferior part of the cerebellar vermis and possibly a small defect of the hiatus tentorium cerebelli.

Subject 5:I.1

The deceased father of II.2 and II.4 (I.1) had a high forehead, facial asymmetry and hearing deficits, suggesting that the *ZIC1* mutation present in his two daughters had been transmitted from him.

WGS500: names and affiliations of authors

Steering Committee: Peter Donnelly (Chair)[1], John Bell[2], David Bentley[3], Gil McVean[1], Peter Ratcliffe[1], Jenny Taylor[1,4], Andrew Wilkie[4,5]

Operations Committee: Peter Donnelly (Chair)[1], John Broxholme[1], David Buck[1], Jean-Baptiste Cazier[1], Richard Cornall[1], Lorna Gregory[1], Julian Knight[1], Gerton Lunter[1], Gil McVean[1], Jenny Taylor[1,4], Ian Tomlinson[1,4], Andrew Wilkie[4,5]

Sequencing & Experimental Follow up: David Buck (Lead)[1], Christopher Allan[1], Moustafa Attar[1], Angie Green[1], Lorna Gregory[1], Sean Humphray[3], Zoya Kingsbury[3], Sarah Lamble[1], Lorne Lonie[1], Alistair Pagnamenta[1], Paolo Piazza[1], Guadelupe Polanco[1], Amy Trebes[1]

Data Analysis: Gil McVean[1] (Lead), Peter Donnelly[1], Jean-Baptiste Cazier[1], John Broxholme[1], Richard Copley[1], Simon Fiddy[1], Russell Grocock[3], Edouard Hatton[1], Chris Holmes[1], Linda Hughes[1], Peter Humburg[1], Alexander Kanapin[1], Stefano Lise[1], Gerton Lunter[1], Hilary Martin[1], Lisa Murray[3], Davis McCarthy[1], Andy Rimmer[1], Natasha Sahgal[1], Ben Wright[1], Chris Yau[6]

[1]The Wellcome Trust Centre for Human Genetics, Roosevelt Drive, Oxford, OX3 7BN, UK

[2]Office of the Regius Professor of Medicine, Richard Doll Building, Roosevelt Drive, Oxford, OX3 7LF, UK

[3]Illumina Cambridge Ltd., Chesterford Research Park, Little Chesterford, Essex, CB10 1XL, UK

[4]NIHR Oxford Biomedical Research Centre, Oxford, UK

[5]Weatherall Institute of Molecular Medicine, John Radcliffe Hospital, Headington, Oxford OX3 9DS, UK

[6]Imperial College London, South Kensington Campus, London, SW7 2AZ, UK
